# Cell-type specific RNA-Seq reveals novel roles and regulatory programs for terminally differentiated *Dictyostelium* cells

**DOI:** 10.1186/s12864-018-5146-3

**Published:** 2018-10-22

**Authors:** Koryu Kin, Gillian Forbes, Andrew Cassidy, Pauline Schaap

**Affiliations:** 1 School of Life Sciences, University of Dundee, Angus, Dundee, DD15EH UK; 2 Tayside Centre for Genomic Analysis, University of Dundee, Angus, Dundee, DD19SY UK

**Keywords:** *Dictyostelium discoideum*, Cell type specific transcriptomics - evolution of soma - cup cells, Small GTPase mediated signalling, *hssA* like genes

## Abstract

**Background:**

A major hallmark of multicellular evolution is increasing complexity by the evolution of new specialized cell types. During Dictyostelid evolution novel specialization occurred within taxon group 4. We here aim to retrace the nature and ancestry of the novel “cup” cells by comparing their transcriptome to that of other cell types.

**Results:**

RNA-Seq was performed on purified mature spore, stalk and cup cells and on vegetative amoebas. Clustering and phylogenetic analyses showed that cup cells were most similar to stalk cells, suggesting that they share a common ancestor. The affinity between cup and stalk cells was also evident from promoter-reporter studies of newly identified cell-type genes, which revealed late expression in cups of many stalk genes. However, GO enrichment analysis reveal the unexpected prominence of GTPase mediated signalling in cup cells, in contrast to enrichment of autophagy and cell wall synthesis related transcripts in stalk cells. Combining the cell type RNA-Seq data with developmental expression profiles revealed complex expression dynamics in each cell type as well as genes exclusively expressed during terminal differentiation. Most notable were nine related *hssA-*like genes that were highly and exclusively expressed in cup cells.

**Conclusions:**

This study reveals the unique transcriptomes of the mature cup, stalk and spore cells of *D. discoideum* and provides insight into the ancestry of cup cells and roles in signalling that were not previously realized. The data presented in this study will serve as an important resource for future studies into the regulation and evolution of cell type specialization.

**Electronic supplementary material:**

The online version of this article (10.1186/s12864-018-5146-3) contains supplementary material, which is available to authorized users.

## Background

Multicellularity evolved at least 10 times independently in most major divisions of eukaryotes [1, 2]. Multicellularity allows cells to not only participate in propagation, but to specialize into roles that promote the propagation of others. Modern animals and plants owe their immense behavioural and morphological complexity to the progressive specialization of such somatic cells. Because somatic cells are in essence altruistic, it remains an intriguing question how cells in early multicellular organisms were enticed to play a purely supportive role.

Dictyostelid social amoebas are an ancient group which is thought to have diverged about six hundred million years ago and includes more than 150 known species [3]. They alternate between unicellular and multicellular stages in their life cycles, with the unicellular forms feeding on bacteria in forest soils. When food is depleted, they undergo multicellular development through aggregation of up to 10^5^ cells, resulting in the formation of a fruiting body. The molecular mechanisms of their development have been explored extensively in a model species, *Dictyostelium discoideum*. Molecular phylogenetic studies in the last 15 years revealed the presence of four major groups in the Dicytostelids [4–6]. Mapping of phenotypic characters onto the phylogeny revealed that most changes in their mode of development occurred during the evolution of a major group 4 which includes *D. discoideum*. Those changes include the use of cAMP as chemoattractant during aggregation, the loss of encystation as a mode of survival, the construction of larger fruiting bodies and the evolution of novel somatic cell types [7, 8].

**Fig. 1:**
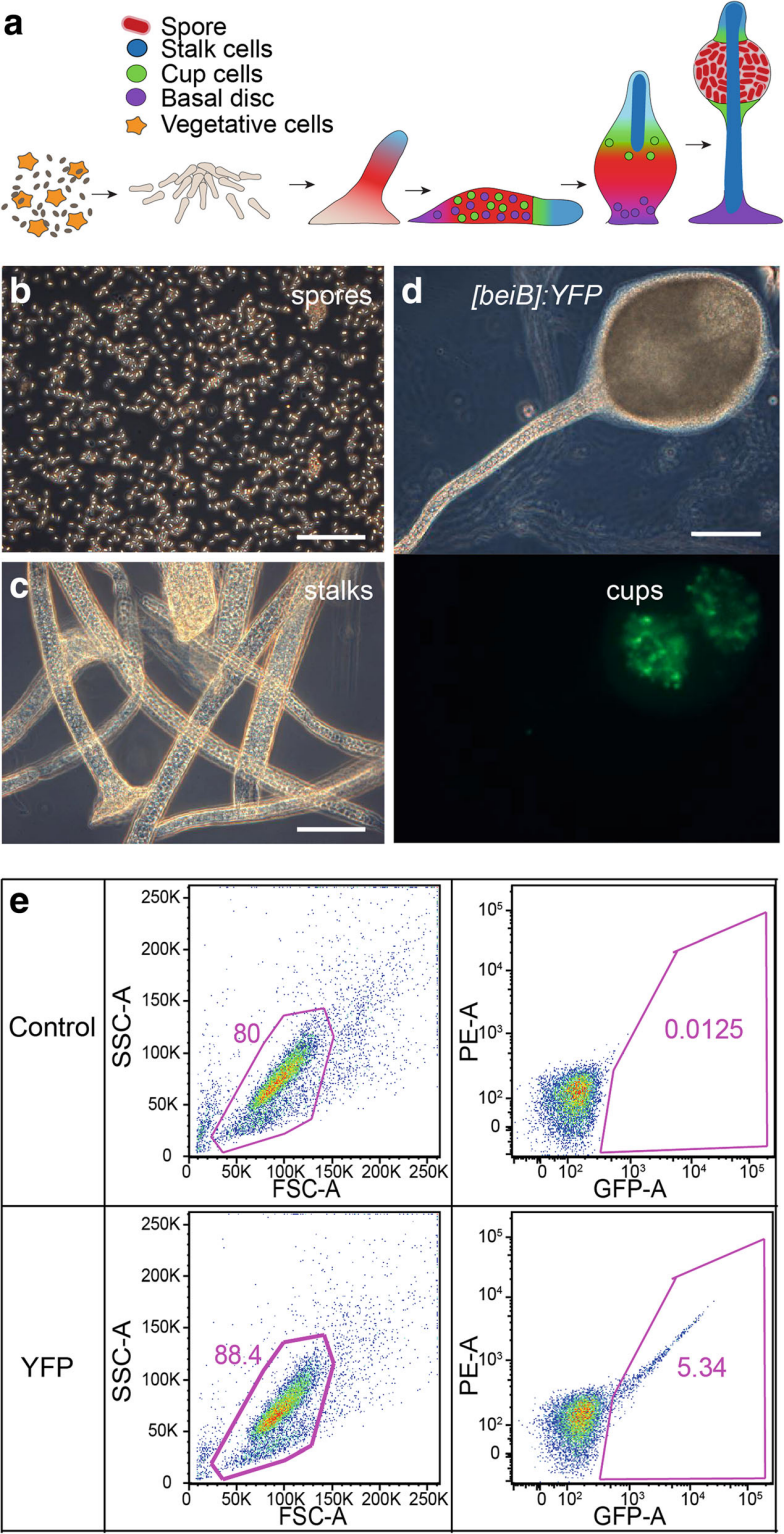
Purification of terminally differentiated cell-types. **a** Schematic drawing of cell differentiation during *Dictyostelium discoideum* development. **b** Phase contrast image of spores isolated from dissociated fruiting bodies by sieving and detergent treatment to lyse amoebas. **c** Fruiting body stalks purified on a Percoll gradient. **d** Cells transformed with a gene fusion of the cup promoter *DDB_G0278537* (*beiB*) and YFP were developed into fruiting bodies and imaged by fluorescence microscopy. Top: Phase contrast. Bottom: YFP fluorescence. Note the less opaque areas in the spore head, corresponding to upper and lower cup regions. **e** An example of FACS experiment with the cellular fraction of dissociated *[beiB]:-YFP* fruiting bodies. Scale bar = 100 μm

The fruiting body of most Dictyostelids consists of two cell types, spores and stalk cells, which correspond to germ cells and somatic cells in metazoans respectively, in the sense that only spores survive and contribute to the next generation. In most Group 4 species, an additional somatic cell type, the cup cells, is present, which are amoeboid cells that demarcate the top and bottom ends of the spore head (Fig. 1a). Some group 4 species also have a basal disc or supporter consisting of stalk-like cells, which anchors the fruiting body stalk to the ground [8]. Like the disc cells, the cup cells are considered to be derived from a subgroup of pre-stalk cells and anterior-like cells (ALCs) in the prespore region [9–12]. Their roles in fruiting body formation have not been much explored, except for a possible role in supporting the elevation of the spore head [10].

A recent study comparing the transcriptomes of wild-type cells and null mutants in diguanylate cyclase, which synthesizes the stalk-inducing factor c-di-GMP [13] revealed both novel stalk genes and a number of genes which are expressed exclusively in the cup cells [14]. Unlike previously recognized cup-expressed genes, most of these genes are not expressed earlier in anterior-like cells, but only very late when spores are maturing in the elevated spore head. These observations imply that cup cells have roles other than lifting the spore head, and indicate the presence of a regulatory program specifically active at the late stage of development.

The transcriptomes of spores and stalk cells cell in the mature *D. discoideum* fruiting body were previously analysed using microarrays with about 7000 cDNAs [15], but cup cells were not yet studied. There have been many RNA-seq based analyses of gene expression during the developmental programme [16, 17], for prestalk and prespore cell populations in the slug stage [16], and even single cell RNA-Seq studies at the early stages of development [18], but our understanding of the repertoires and regulation of cell type specific genes during terminal development is limited.

In this study, we isolated cup cells by fluorescence activated cell sorting (FACS) using a cup specific marker gene and collected the RNA-Seq data of cup cells, spores, stalk and feeding amoebas. Our major goal is to better understand the nature of cup cells and to determine their relationship to spores and stalks. Our study also led to better characterization of the cell type specific regulatory programs that act in late *D. discoideum* development.

## Results

### Cell type isolation

Vegetative cells were harvested while growing in exponential phase. Spore, stalk and cup cells were isolated from fruiting bodies at 24 h since the onset of development. Spore cells were isolated by sieving dissociated fruiting bodies through nylon mesh and removing amoeboid cells in the flow-through with detergent. (Fig. 1b). For stalks, removal of adhering amoeboid cells with detergent had detrimental effects on the quality of the isolated RNA, and we therefore centrifuged stalks, collected from the top of the nylon mesh, through a Percoll gradient, which left amoeboid cells and spores at the bottom of the tube and the stalks at a density interphase. The collected stalks contained only a negligible amount of amoebas or spores (Fig. 1c).

Cup cells could not be isolated by physical separation methods only. We therefore introduced YFP linked to the *DDB_G0278537* cup specific promoter into wild type cells and confirmed that YFP fluorescence was restricted to the cup cells (Fig. 1d). (*DDB_G0278537* was initially named *cupB*, but as the CUP acronym was already used for *Dictyostelium* Ca^2+^-upregulated genes, we replaced *cup* with *bei*, Chinese for cup, for this and three other late cup-specific genes [13], see also Additional File 1: Table S6). After dissociating fruiting bodies into single cells and stalks, and removing stalks by sieving, the remaining suspension, which consisted of spores and amoeboid cells, was subjected to Fluorescence Activated Cell Sorting (FACS). About 3–5% of cells in the single cell suspension were found to be YFP positive (Fig. 1e) and were collected, which resulted in 3 × 10^4^ to 10^5^ cells for each replicate. When observed under the microscope, spore cells were not totally absent in the FACS sorted population, but the fraction of spore contamination in each replicate was small (2.2–7.4%). The chance of isolating RNA from contaminating spores is expected to be minimal due to the low efficiency of spore RNA isolation without mechanical wall disruption. RNA was purified from three independent isolates of cup cells, spores, stalks and vegetative cells and subjected to Illumina NextSeq paired-end sequencing. Due to the small number of cells available for cup cells, TRIZol was used for increased yield of RNA to isolate RNA from cup samples, while RNA from other samples was isolated with spin columns. We found no evidence that the use of two different techniques for isolating RNA introduced systematic biases in downstream RNA-Seq analyses, such as sample clustering according to RNA isolation method.

### General RNA-Seq statistics and hierarchical clustering

On average, 29 million (ranged from 24 million to 42 million) 75 bp paired-end RNA-Seq reads were obtained from each replicate (See Additional file 2 for full dataset and Additional file 1: Table S2 for summary statistics). The overall mapping rates for the cup cell samples were almost 20% lower than those for the other samples, due to the presence of reads derived from YFP that are not mappable to the *Dictyostelium* genome. When excluding these reads, the mapping rates for cup samples were still 6–7% lower than those of other cells, possibly due to slightly lower RNA quality.

**Fig. 2:**
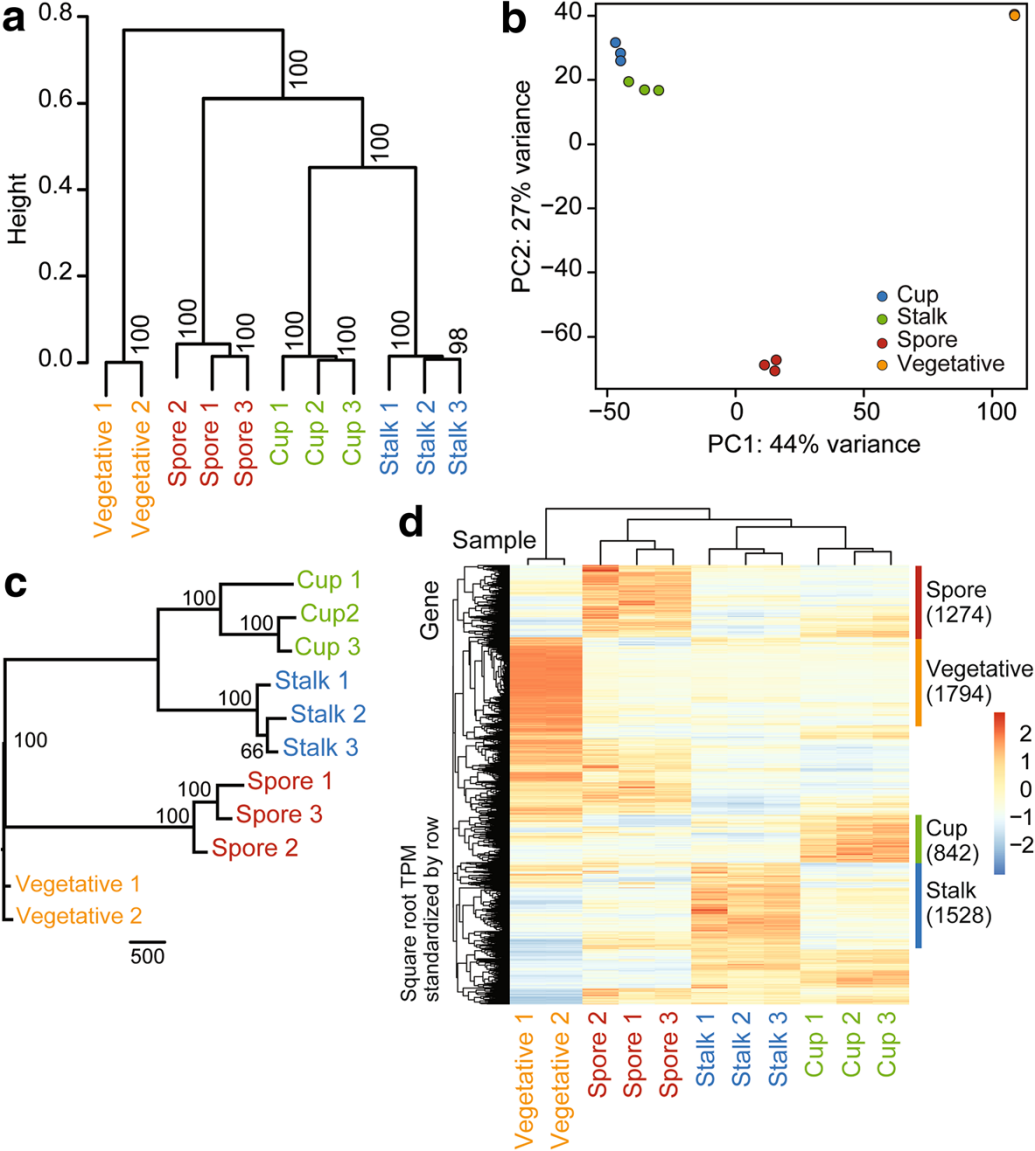
Bioinformatic analysis of RNAseq data from purified cell types. **a** Hierarchical clustering. The dendrogram of all RNA-Seq samples obtained with pvclust is shown with the bootstrap values on each node. Spearman’s correlation was used as a distance metric with the average linkage algorithm. **b** Principal component analysis (PCA). PCA plot of all RNA-Seq samples. Replicates of the same cell type are indicated by the same color as shown in the legend. **c** Maximum parsimony tree of all RNA-Seq samples with bootstrap values on each node. Scale bar corresponds to 500 hundred changes in binary gene expression. **d** Heat map of gene expression profiles shown with gene-wise (row) and sample-wise (column) clustering dendrograms. The colour corresponds to the square root of standardized TPM values (i.e., scaled by the standard deviation and centred to the mean) by row as shown in the legends on the left. Putative cell type specific gene clusters are shown on the right with the number of genes which belong to each cluster in parenthesis

We first analysed the overall gene expression patterns by unsupervised clustering methods. The result of the hierarchical clustering with pvclust is shown in Fig. 2a. It shows that cup cells form a cluster with stalk cells with high bootstrap support. The cup-stalk cluster further forms a cluster of developmental cell types with spore cells in exclusion of vegetative cells. The topology of the dendrogram remained unchanged when we used different metrics and linkage algorithms for clustering (data not shown). The closeness of gene expression patterns between cup cells and stalk cells was also evident from Principle Component Analysis (PCA) (Fig. 2b), where the cup and stalk cell replicates are located close to each other.

It was previously shown that maximum parsimony phylogenetic reconstruction can be used to infer relationships between cell types from RNA-Seq data [19]. After transforming the quantitative RNA-Seq expression data into binary data, the maximum parsimony algorithm was applied, using vegetative cells as the out-group. This method also yielded a well-resolved tree, which supports the sister group relationship between cup cells and stalk cells (Fig. 2c).

Gene-wise hierarchical clustering was combined with sample-wise clustering to visualize cell-type specific gene expression clusters on a heatmap (Fig. 2d). In the heatmap, there are clearly clusters of genes which are expressed in a cell type specific manner. The lists of genes in the clusters were combined with differential gene expression analyses to produce conservative estimates of cell type specific genes as described below.

### Differentially expressed genes and GO term analysis

In order to identify genes which are specifically upregulated in each cell type, the transcriptome of each cell type was compared with those of other cell types. Since the transcriptome of vegetative cells was very different from those of the developmental cell types, it was excluded when a developmental cell type was compared with the other developmental cell types. The overall results are summarised in Table 1. The list of genes that were up-regulated in each cell type was further compared to the genes which belonged to a cell type specific cluster as shown in Fig. 2d. We took the intersection of the two lists and used the result as a conservative estimate of cell type specific genes in the late stage of *D. discoideum* development (Table 1).

**Table 1:** Differentially expressed genes

Cell Type	Up-regulated	Down-regulated	Total in up-regulated cluster	Up-regulated intersection
Cup	1639	1454	842	742
Stalk	1561	1405	1528	757
Spore	1925	2036	1274	797
Vegetative	2472	2681	1794	1587

EdgeR was used to compare gene expression levels in cup, stalk, and spore samples with those in stalk+spore, cup+spore, cup+stalk samples, respectively, to determine the number of differentially expressed genes in each cell type with FDR < 0.05. Vegetative cells were compared with cup+stalk+spore. Up-regulated genes were further compared to genes found in cell type specific clusters identified by gene-wise hierarchical cluster analyses as shown in Fig. 2d.

**Fig. 3:**
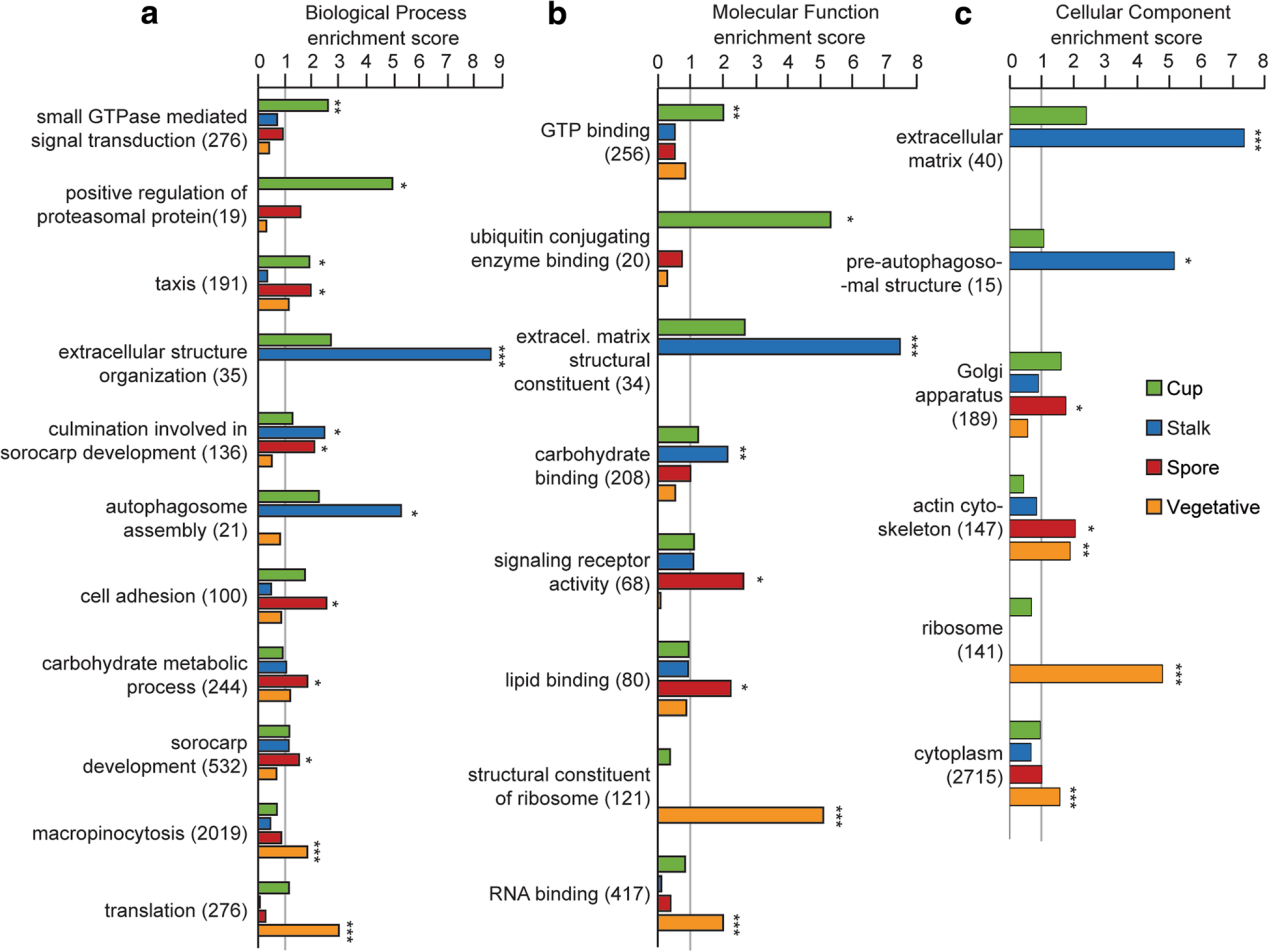
GO term enrichment analysis. GO term enrichment for each cell type specific gene list was analysed using topGO [60] and GO terms with statistically significant enrichment were examined. Since GO terms are organized hierarchically, the full list (see Additional file 3) contains many hierarchically related GO terms. Here we show hierarchy-representative GO terms that are enriched in each set of cell-type specific genes in the GO categories Biological Process (**a**), Molecular Function (**b**) and Cellular Component (**c**) Enrichment scores for cup, stalk, spore, and vegetative cell specific genes are presented as green, blue, red and amber bars, respectively. Single, double and triple asterisks signify *p*-values smaller than 0.01, 10^− 4^ and 10^− 10^, respectively. Numbers between parentheses represent the total numbers of genes annotated with each GO term in the current dataset

To gain insight into functions specific to each cell-type, we first performed Gene Ontology (GO) term enrichment analyses of the cell-type specific genes. The results of the complete analyses can be found in Additional file 3, while Fig. 3 shows representative enriched GO terms in each of the GO categories Biological Process (BP), Molecular Function (MF) and Cellular Component (CC). Since GO terms have hierarchical structures, we selected the more general terms for comparisons between cell types, while GO terms comprising fewer than 10 genes are excluded to avoid spurious results.

Cup cells show the most significant enrichment in genes involved in signal transduction, especially small G-proteins, as indicated by the GO BP terms such as “small GTPase mediated signal transduction” and GO MF terms “GTP binding”. Further investigation of the expression levels of the signal transduction genes across cell-types highlights that many of the G-proteins and other signal transduction proteins, such as *rapA*, *racE*, *gpaB* and *plc* are involved in “taxis” (Additional file 1: Figure S1a), another GO term enriched in the cup gene set. Cup cells may also be actively catabolizing proteins through ubiquitination as inferred from the enriched BP terms “positive regulation of proteasomal ubiquitin-dependent protein catabolic process” and MF term ubiquitin conjugated enzyme binding.

Stalk cells show by far the strongest enrichment in genes involved with the formation of extracellular matrix. Many genes in this category encode uncharacterized proteins with inferred roles in matrix formation due to the presence of cellulose binding domains (Additional file 1: Figure S1b). GO terms related to autophagy such as “autophagosome assembly” (GO:0000045) and pre-autophagosomal structure (GO:0000407) are also enriched.

Spores show enrichment of GO BP terms such as “cell adhesion” (GO:0007155), “carbohydrate metabolic process” (GO:0005975) and GO MF terms such as “lipid binding” (GO:0008289) and “signalling receptor activity” (GO:0038023). The signalling receptors include the histidine kinases *dhkF*, *dhkJ* and *dhkH*, as well as the endosomal transmembrane proteins *phg1A*, *phg1B and phg1C,* and the glutamate receptor like proteins *grlA* and *grlC* (Additional file 1: Figure S1c). GrlA is known to be required for spore maturation [20], but the roles of the other “signalling receptors” are unclear. GO CC terms showed slight enrichment for spore genes in “Golgi apparatus” (GO:0005794) and significant enrichment in “actin cytoskeleton” (GO:0015629), possibly related to the formation of actin rods in spores [21].

Vegetative cells show enrichment of genes related to feeding and growth (Fig. 3), represented by highly significant enrichment of GO BP terms such as “macropinocytosis” (GO:0044351) and “translation” (GO:0006412).

### Metabolic pathway analysis

**Fig. 4:**
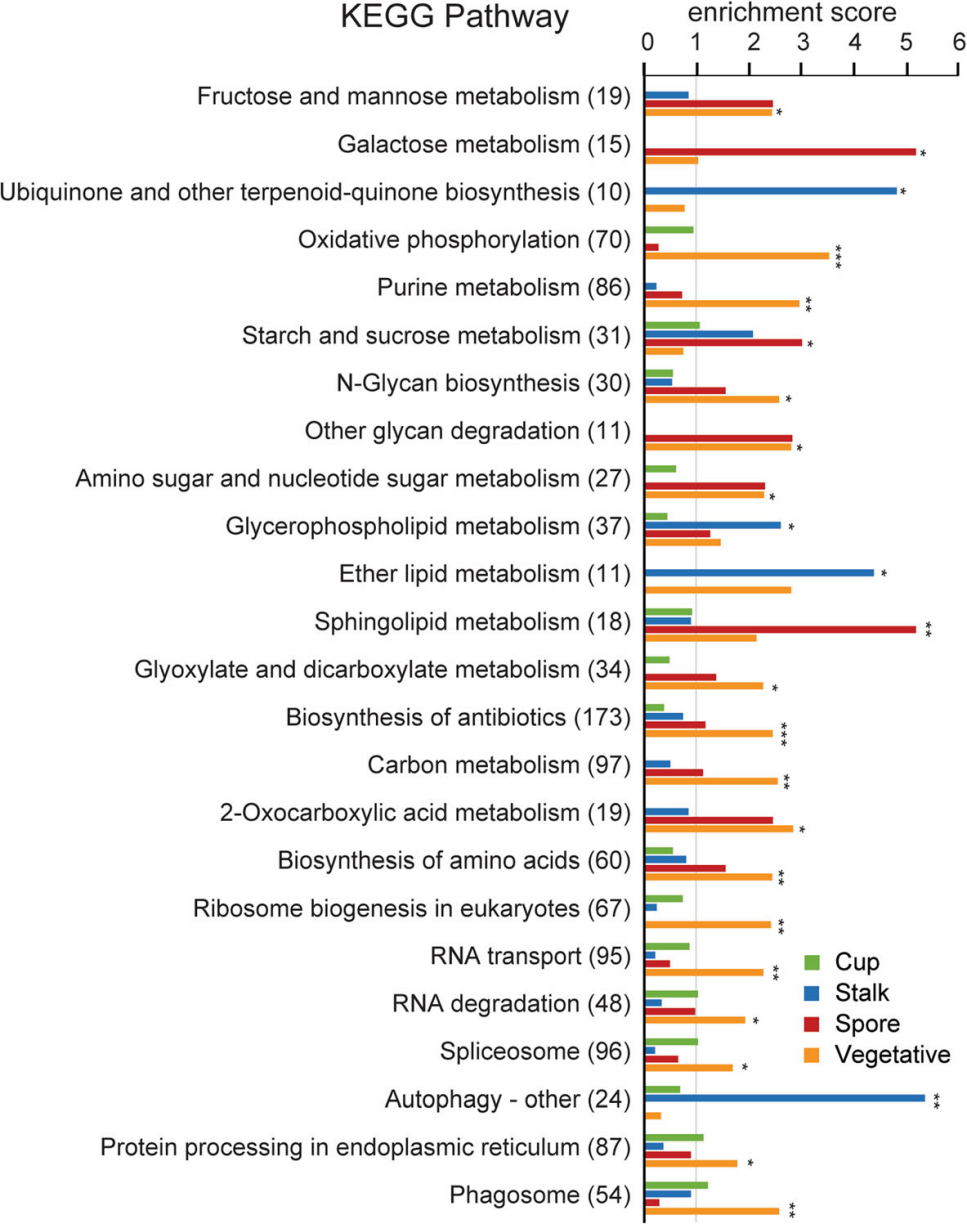
KEGG pathway analysis. Cell type specific genes were associated with metabolic pathways according to KEGG pathway database (http://www.genome.jp/kegg/pathway.html). Enrichment scores were calculated by dividing the actual numbers of cell type specific genes associated with a pathway by the expected number of genes to be observed, where expected number was calculated as the number of cell type specific genes times the proportion of genes associated with the pathway in the genome. *P*-values were calculated with Fisher’s exact test. Single asterisk (*) means *p* < 0.05, double asterisk (**) *p* < 10^− 4^, triple asterisk (***) < 10^− 10^

To gain additional insight into the specific functions of cup, stalk and spore cells, we performed an analysis of specific metabolic pathway enzymes enriched in each of the cell types using the KEGG pathway Database [22] (http://www.genome.jp/kegg/pathway.html) with the Search&Color Pathway option. The full result of the mapping is shown in Additional file 4, sheet 1, with the pathway enzymes that are enriched in each cell type shown in sheet 2. Figure 4 shows summary results displaying only pathways that differ considerably between cell types and contain at least 3 enzymes in one of the terminal cell types. The analysis shows that spores are significantly enriched with enzymes of several sugar metabolic pathways (statistically significant: galactose, starch/sucrose, amino/nucleotide sugar; marginally significant: fructose/mannose and amino/nucleotide sugar) and sphingolipid pathways, but specifically poor in enzymes of ribosome biogenesis and autophagy. The enriched pathways probably reflects sugar utilization in the cellulose and glycoprotein-rich spore wall and/or the deposition of polysaccharide storage compounds. Stalk cells are enriched in enzymes of quinone synthesis, autophagy, ether lipid and glycerophospholipid metabolism, but poor in pyruvate, glyoxylate/ dicarboxylate and amino/nucleotide sugar metabolism and oxydative phosphorylation enzymes, indicating the preponderance of catabolic processes and decline in ATP generating processes in stalk cells. Cup cells do not show significant enrichment of particular pathways, while conversely vegetative cells show enrichment of most pathways except autophagy and ubiquinone biosynthesis.

The spectrum of enriched GO terms and KEGG pathways associates cup cells with active signal transduction and motility, stalk cells with autophagy and extracellular matrix formation, and spores with sugar and sphingolipid metabolism. Not surprisingly, feeding cells are most metabolically active and have no need for autophagy.

### Developmental expression profiles of cell-type specific genes

**Fig. 5:**
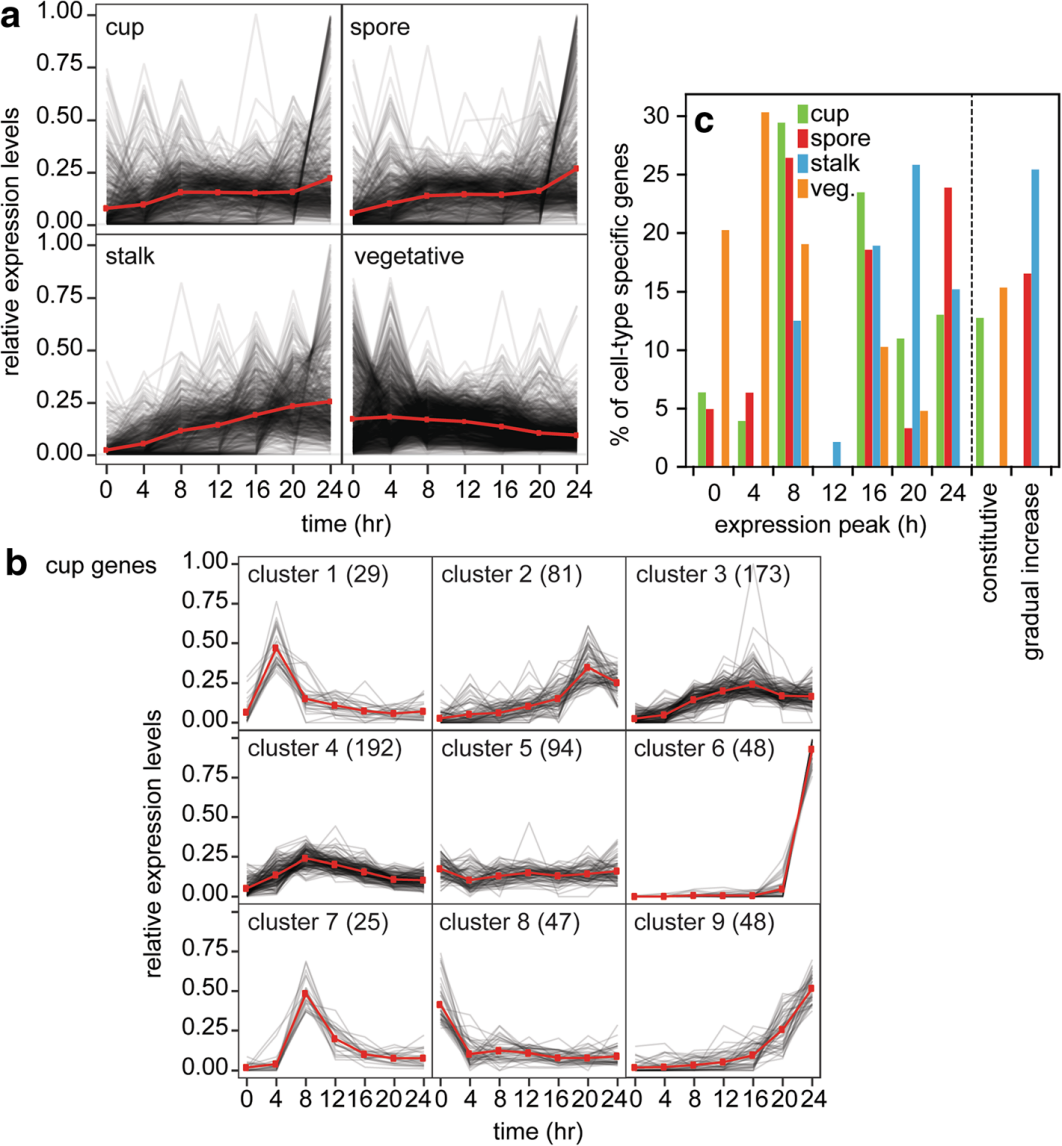
Developmental regulation of cell type specific genes expression. **a** Expression profiles of all cell-type specific genes. The gene expression level of each gene at each time point, as retrieved from [16] is expressed as fraction of the sum of its expression at all time points. Each grey line represents the expression profile of a single gene. The red line represents the expression average of all genes. **b** Cluster analysis of cup genes*.* The developmental expression profiles of cup genes were subdivided into 9 clusters by k-means clustering. The number of genes which belong to each cluster is shown in parentheses. The average trend is shown in red points and lines. See Additional file 1: Figure S2 for cluster analysis of spore, stalk and vegetative genes. **c** Cluster analysis summary*.* Most clusters showed peak expression at a single time point, or alternatively constitutive expression or a gradual increase. For each cell type, the number of genes with peak expression at 0, 2, 4 etc., hours or with constitutive or gradually increasing expression were expressed as percentage of the total number of specific genes for that cell type

To gain insight into the stage(s) at which stalk, cup and spore genes are induced, we analysed their developmental expression profiles using published developmental transcriptome data [16]. Figure 5a shows that the expression levels of stalk genes increase steadily toward the end of fruiting body formation at 24 h, with some genes peaking at 16 or 20 h. The expression levels of spore and cup genes also increase on average toward 24 h, with some subsets of genes showing peaks of expression before and during aggregation. Notably, there are sets of cup and spore genes, which are almost exclusively expressed at 24 h. Conversely, most growth specific genes show peak expression at 0 or 4 h and are then progressively downregulated.

In order to identify groups of genes with similar gene expression profiles, we used k-means clustering to classify each gene. K-means clustering assumes a certain number of clusters in the data, assigns each gene into a cluster, and tries to minimize the variation within each cluster through iterative reassignments of genes into clusters. The number of clusters was set to nine for this data set, as it separates genes with different expression profiles well without subdividing them too much. Figure 5b shows the nine types of developmental expression profiles for cup-specific genes and in Additional file 1: Figure S2 those for spore, stalk and vegetative genes. Full results can be found in Additional file 5.

The clusters mostly differ in the time of peak expression of the genes or alternatively show constitutive or slowly increasing expression. For each cell-type we calculated the percentage of genes with peak expression at a specific time of development or with constitutive or slowly increasing expression (Fig. 5c). Almost one-third of spore and cup specific genes showed peak expression at 8 h, followed by 24 and 16 h for spores and 16 and 24 h for cup cells. About a quarter of stalk-specific genes increased slowly upon starvation to reach a plateau at 8 h, while another quarter showed peak expression at 20 h, followed by smaller sets peaking at 16 h and 24 h. Vegetative genes showed peak expression at 4 h followed by 0 h. Evidently, a large proportion of spore and cup specific genes are already induced during or just after aggregation (8 h) or in slugs (16 h), while many stalk-specific genes are induced just after aggregation or in early fruiting bodies (20 h). We also used more stringent thresholds for calling genes “cell type specific” to test the robustness of these results. Even when using a more stringent set, cell type specific genes with early gene expression peaks were still present (Additional file 1: Figure S3). This suggests that their presence was not artefactual, but likely reflects that cell type specificity can also arise by genes being down-regulated in all but one cell type.

All three terminal cell types show a set of genes that is almost exclusively expressed at 24 h (Clusters 6, 1 and 7 in Fig. 5b, Additional file 1: Figure S2a and b, respectively). These genes can be considered to most uniquely represent the terminal cell types. Many of these genes have no proper gene names or known functions, but there are some notable exceptions. The late spore genes include germination factors, such as *celA*, *celB*, *gerA*, *gerB*, and *gerC*, as well as *iptA*, which encodes isopentenyltransferase, an enzyme essential for the synthesis of the germination inhibitor, discadenine. The late stalk genes include *staA*, a previously identified marker for terminal stalk cell differentiation [23].

The cup-specific late expressed genes contained many members of the hssA/7E/2C family [24], which are also the most highly expressed genes in the set. The *hssA* genes are around 213 bp in size, with a 13 bp first exon and ~ 200 bp second exon. A phylogenetic tree of all *hssA* genes reveals a closely related subset, which is almost exclusively expressed in cup cells at the end of fruiting body formation (Additional file 1: Figure S4).

### Expression of prestalk/prespore genes in stalk, spore and cup cells

To understand the relationship between the genes specifically expressed in terminally differentiated spore, stalk and cup cells and genes expressed in their precursors, i.e. the prespore and prestalk/anterior-like cells (ALC), we compared the cell type specific gene lists with the published lists of genes enriched in prespore and prestalk/anterior-like fractions that were separated on Percoll density gradients (which do not discriminate between prestalk and anterior-like cells [25, 26]).

**Fig. 6:**
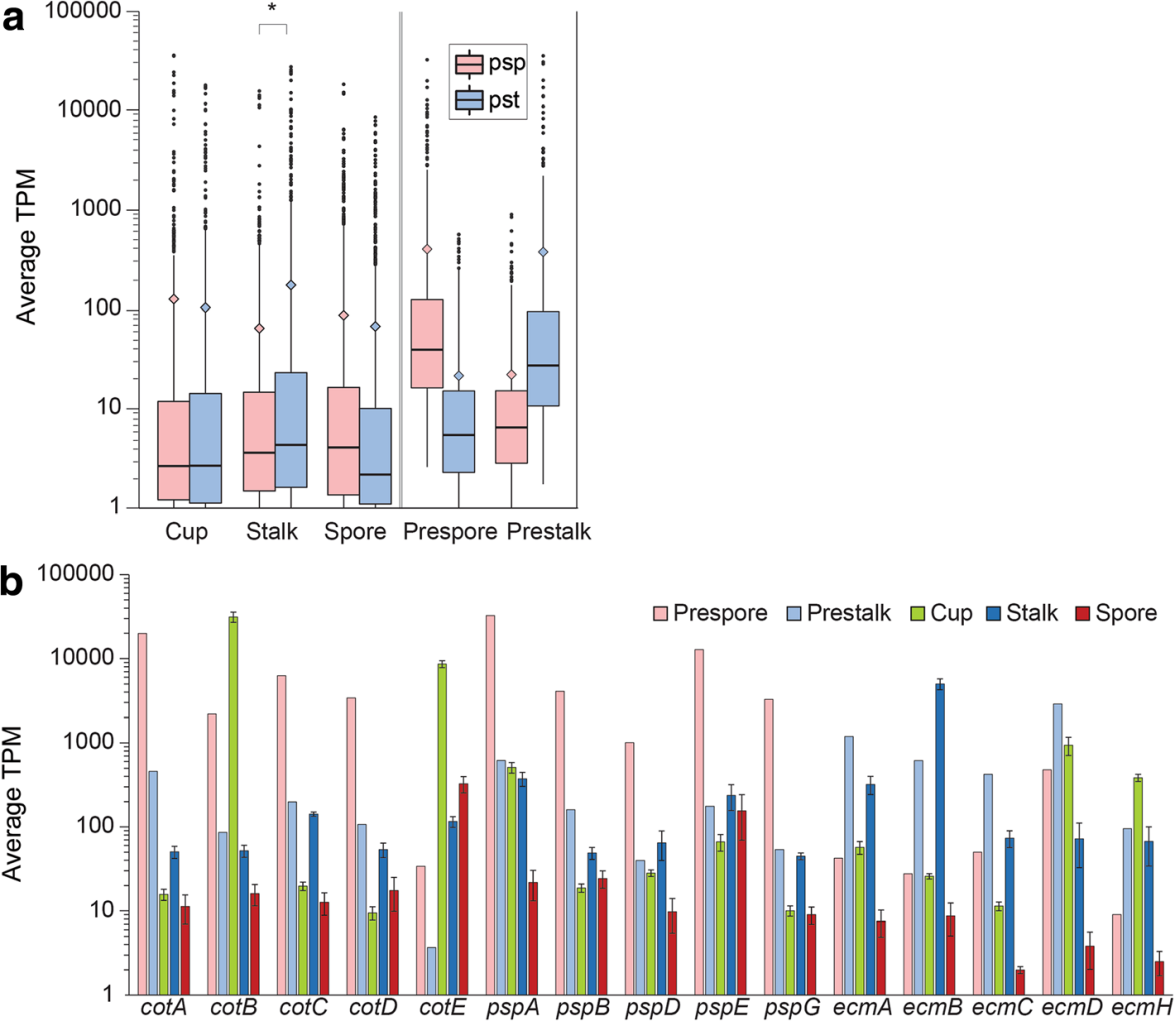
Prespore and prestalk gene expression in terminally differentiated cells. **a** Boxplots of “prespore” (pink) and “prestalk” (light blue) gene expression in each terminal cell type. The set includes 840 prespore genes and 913 prestalk genes identified in [16]. For comparison, the expression levels in prespore and in prestalk cells from the original data are also shown. The middle lines correspond to the medians and filled diamonds correspond to the means. The difference in the mean of expression levels of prespore and prestalk genes in stalk, but not in spore and cup cells, is statistically significant (*p* < 0.01, shown by asterisk). The lower and upper hinges in the boxplots correspond to the 25 and 75 percentiles, respectively. Outliers are shown as filled black circles. **b** Expression levels of select prespore and prestalk genes in precursor and mature cell types. Error bars represent 1 standard error (SE) of the mean from three replicates

We first investigated how the prespore and the prestalk/ALC genes are expressed in our dataset. On average, stalks express prestalk/ALC genes more than prespore genes (*p* < 0.01), while spore and cup cells express prespore and prestalk genes at similar levels. The distribution of expression levels is highly skewed towards low expression. Only a small portion of the prestalk and prespore genes are highly expressed in terminal cell types with the majority of genes having Transcripts Per Million (TPM) less than 3, as seen by huge differences between medians and means (Fig. 6a). This suggests that many prespore or prestalk/ALC genes are downregulated in terminally differentiated cells. Figure 6b shows expression levels of known prespore or prestalk genes in cup, stalk and spore cells. Notably, cup cells express some prespore genes, such as the spore coat genes *cotB* and *cotE* at very high levels. In contrast, these and many other spore coat genes and prespore markers are not highly expressed in spores. Stalk cells express many *ecm* genes at high levels as expected.

### Expression of transcription factors

We next examined the expression patterns of transcription factors (TF), which are major determinants of cell type identity. Since there is no definitive list of TFs in the *D. discoideum* genome, we first searched for all the genes with DNA binding domains by Interpro domain search and identified 247 putative TF genes. Using this list, we further identified 29, 23, and 37 TF genes which are specifically upregulated in cup, stalk and spore cells respectively (Additional file 1: Tables S3-S5). Many of these TFs have no known function, but there are also several TF genes which have been well characterized. TF genes that are at least 3-fold enriched in cup, stalk or spore cells with at least 50 transcripts per million are shown in Table 2.

**Table 2:** Cell-type enriched transcription factors

Gene	TF family	Transcripts Per Million			Fold-change
		Cup	Stalk	Spore	
cup-enriched					
*srfA*	MADS-box	2464	1532	134	3.0
*mybE*	myb-type	903	52	164	8.4
*bzpH*	basic-leucine zipper	146	27	20	6.2
*DDB_G0286351*	cudA-like transcription factor	101	40	0	5.0
*DDB_G0278761*	NF-X1-type zinc finger	94	4	14	10.6
*bzpD*	Basic-leucine zipper	82	32	16	3.4
stalk-enriched					
*DDB_G0291348*	zinc cluster transcription factor	325	1229	4	7.5
*bzpF*	basic-leucine zipper	402	902	9	4.4
*gtaG*	GATA-zinc finger	250	472	3	3.7
*gtaC/dimC*	GATA-zinc finger	59	243	6	7.5
DDB_*G0284591*	C2H2-type zinc finger	95	164	16	3.0
*cbfB*	cbf/jumonji-type	1	86	1	99.9
*hbx14*	homeobox	5	66	0	13.0
spore-enriched					
*dr1*	histone-like transcription factor	15	37	402	15.6
*DDB_G0278179*	myb domain	128	48	354	4.0
*DDB_G0274691*	C2H2-type zinc finger	57	12	220	3.2
*DDB_G0271886*	PHD zinc finger, bromodomain	16	9	165	6.5
*gtaH*	GATA-zinc finger	31	38	148	4.3
*hbx10*	Homeobox	6	1	91	12.5
*gtaN*	GATA-zinc finger	1	2	79	22.5
*cdc5l*	myb-type, CDC5L ortholog	9	8	61	3.5
*DDB_G0267638*	myb and SNF2-related	7	10	59	3.4

The set of 247 *D.discoideum* putative transcription factors was analysed using edgeR to identify factors with enriched expression in cup, stalk or spore cells. See Additional file 1: Tables S3-S5 for the full list. All TFs with at least 3-fold enrichment and 50 transcripts per million are listed above. Fold change here refers to the expression level in a particular cell type relative to the average of two other cell types.

Cup cells express *mybE* significantly more than other cell types, which is consistent with its role in ALC formation [27], but also *srfA* [28], required for proper spore formation and four TFs with unknown roles. Stalk cells specifically expressed *gtaC* with known roles in aggregative and prestalk gene expression [29, 30], *bzpF* required for spore formation [31] and five other TFs with unknown roles. No TFs known to be required for spore differentiation, such as *srfA*, *bzpF*, *stkA* or *spaA* [28, 31–33] are upregulated in spores, while *cudA*, required for both spore and stalk formation [34] is most strongly expressed in stalk and cup cells. Instead, the most highly expressed TF gene in spores is *dr1*, a well-known general repressor of transcription conserved from yeast to humans [35, 36]. *Dr1* forms a heterodimer with *drap1* in a protein complex called negative cofactor 2 (NC2) which inhibits transcription initiation by TATA box binding protein (TBP). *Drap1* is also specifically upregulated in spores along with *dr1*, albeit at a lower level (Additional file 1: Table S5). In addition, spores express six other TFs with unknown roles.

### Developmental origin of cup cells

**Fig. 7:**
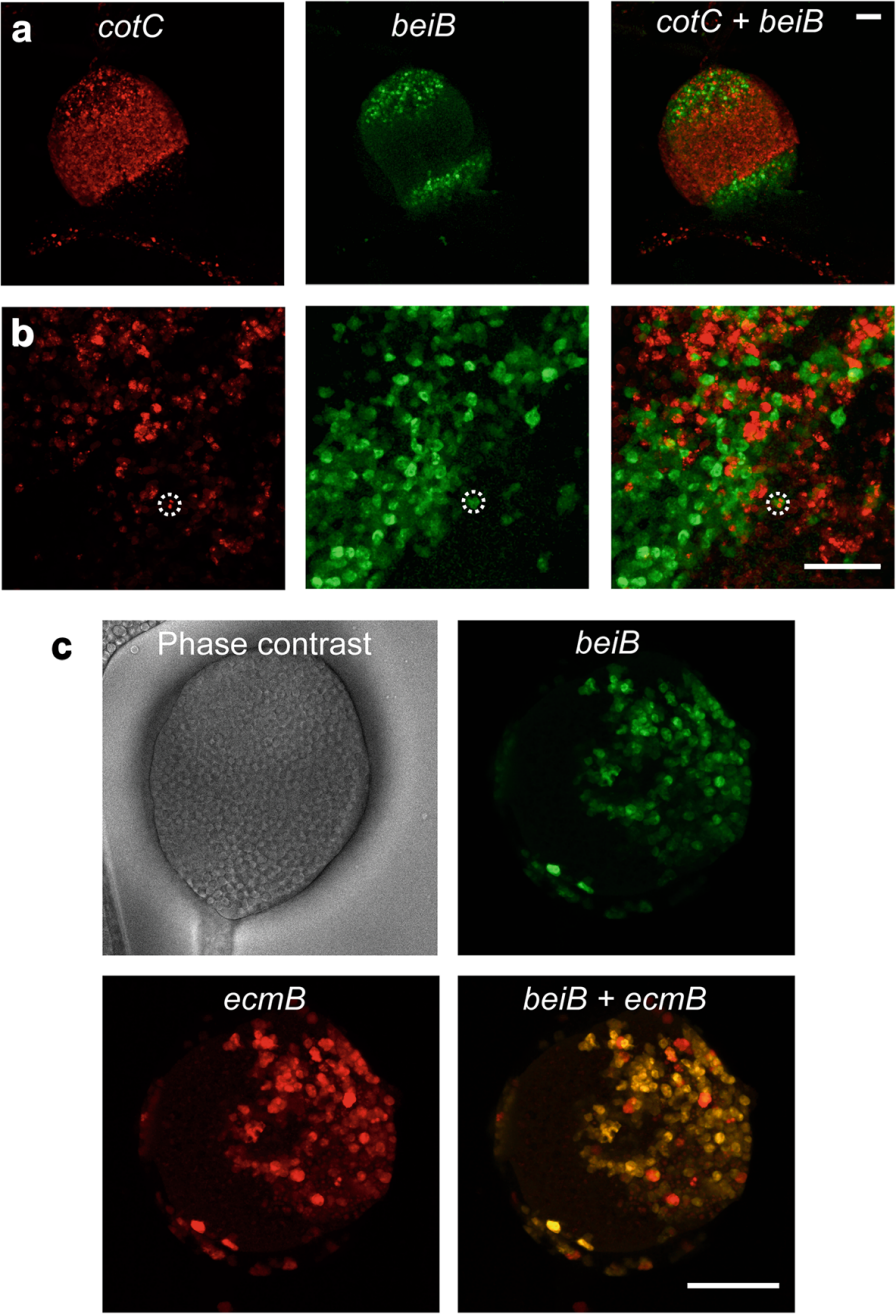
Co-expression patterns of late cup and prestalk or prespore marker genes. Cells were co-transformed with fusion constructs of *RFP* and the *cotC* or *ecmB* promoters*,* and of *YF*P with the *beiB* promoter. Co-transformed cells were developed to fruiting bodies on a thin layer of agar. A small portion of the agar was excised for imaging of spore heads using a Leica TCS SP8 confocal microscope detecting RFP or YFP fluorescence. **a**, **b** Spore heads co-expressing [*cotC*]*:RFP* and [*beiB*]*:YFP*. Left and centre panels show RFP and YFP fluorescence, respectively, and in the right panel RFP and YFP fluorescence are superimposed. **a** intact spore head, **b** squashed spore head at higher magnification showing the absence of RFP and YFP co-localization, except for one cell (marked by dashed circle). **c** Spore head showing co-expression [*ecmB*]*:RFP* and [*beiB*]*:YFP*. The first panel is a phase contrast image of the spore head. The remaining panels show [*ecmB*]*:RFP*, *[beiB]:YFP* and superimposed fluorescence images as indicated. Note that there is almost complete overlap between *ecmB* and *beiB* expression. Scale bar = 50 μm

The earlier finding that many cup genes are only expressed late in the spore head [14] and that a number of prespore genes are highly expressed in cup cells (Fig. 6b) suggests that prespore cells transdifferentiate into cup cells during late culmination and that late cup cells are a different population from cup cells derived from ALCs. To test this possibility, we examined the possible co-localization of prespore, prestalk/ALC and cup cell markers at the end of culmination (20-24 h) using cells cotransformed with the late cup promoter *beiB* fused to YFP and the spore promoter *cotC* or the prestalk/ALC promoter *ecmB* [37] fused to RFP. Figure 7a shows that the expression pattern of *cotC* in the spore head is quite distinct from that of *beiB*, with *beiB* flanking *cotC* and almost no colocalization in the same cells. Occasionally, *cotC* could be detected in *beiB* positive cells (Fig. 7b, dashed circle), but these cells only represent a very small fraction in the whole population. On the other hand the expression of *ecmB* strongly overlaps with that of *beiB* (Fig. 7c), indicating that not only the early but also the late cup cells are likely derived from ALCs.

### New cell type specific marker genes

**Fig. 8:**
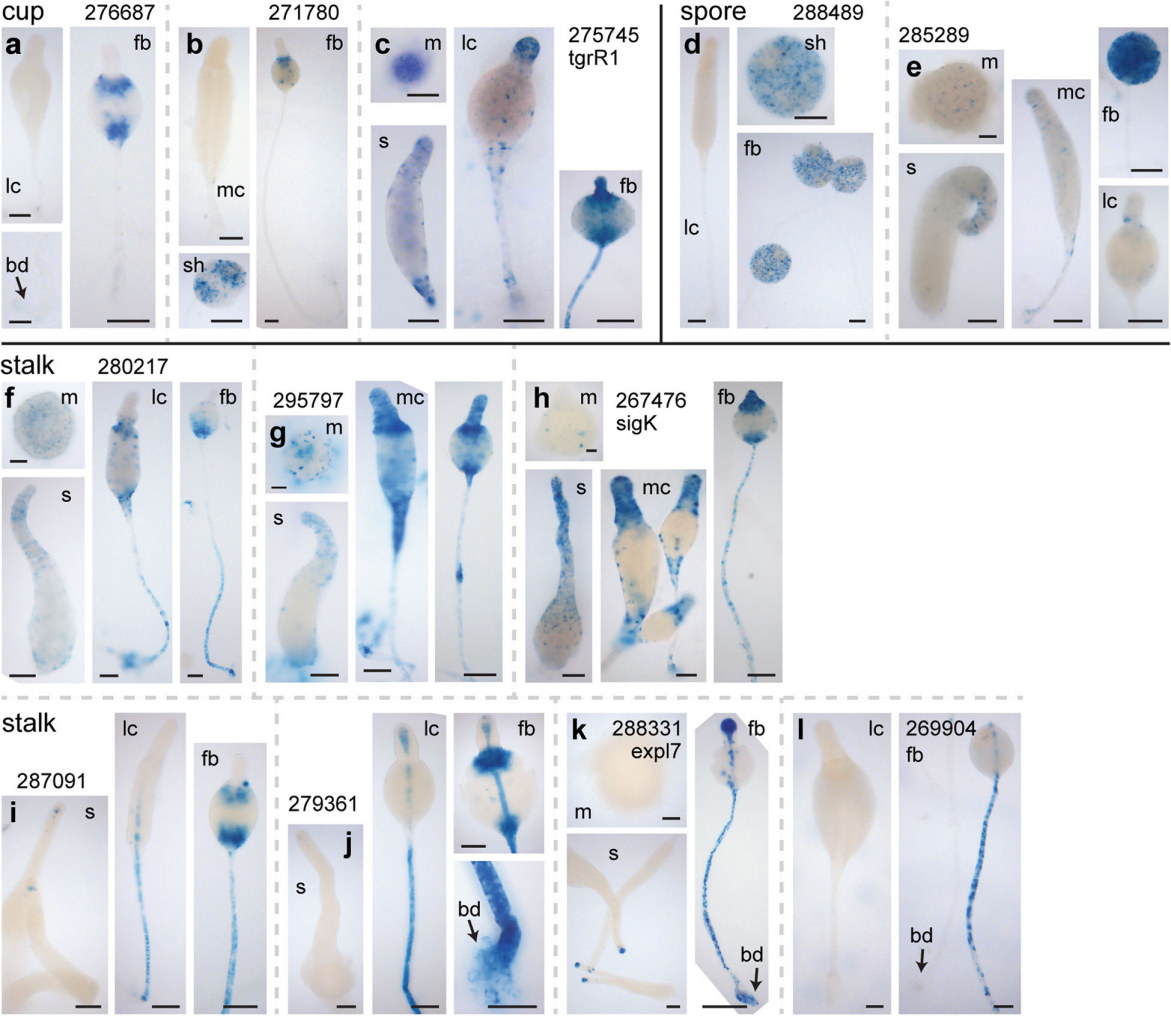
Expression patterns of cell-type specific genes identified by RNA-Seq**.** Wild-type cells, transformed with *lacZ* fused to the promoters of 3 cup genes, 2 spore genes, and 7 stalk genes were developed, fixed, and stained with X-gal at different developmental stages, with the same staining period used per construct for each stage. m: mound; s: slug; mc and lc: mid and late culmination; fb: fruiting body, sh: spore head; bd: basal disc. Developmental stages that are not shown were devoid of staining. **a**-**c** Cup genes *DDB_G0276687*, *DDB_G0271780* and *tgrR1*. **d**, **e** Spore genes *DDB_G0288489* and *DDB_G0285289*. **f**-**l** Stalk genes, *DDB_G0280217*, *DDB_G0295797, sigK, DDB_G02787091*, *DDB_G0279361, expl7* and DDB_G0269904. Bars: 50 μm, gene identifiers are shown without DDB_G0 prefixes

To confirm that the genes with highly-enriched expression in the purified cell types are indeed specific for this cell type and to identify novel cell type markers, we fused the promoters of a number of well-expressed cup, stalk and spore genes to the *lacZ* reporter gene. We next monitored the expression patterns of the genes in developing structures by β-galactosidase histochemistry. The cup genes *DDB_G0276687*, an hssA/7E/2C related gene, and *DDB_G0271780*, a putative alcohol dehydrogenase were both expressed very late in the spore head in the upper- and lower cup (Fig. 8a, b), but not in the basal disc. Expression of these genes occurs during and after maturation of the spores, and is similar to the expression pattern of the two cup genes *DDB_G0276063* (*beiA*) and *DDB_G0278537 (beiB)* [14] that instigated the current study. The third cup gene *tgrR1,* encoding a transmembrane protein, was expressed in cells scattered in mounds and throughout slugs with more pronounced expression at the slug anterior and rear-guard. During culmination, expression became evident in the stalk and basal disc, but was strongest at the tip. Expression in the upper and lower cup only appeared in the late spore head (Fig. 8c).

The spore gene *DDB_G0288489* showed only sporadic expression before fruiting body completion and was then only expressed in spores (Fig. 8d). A second spore gene *DDB_G0295289*, was expressed weakly in the prestalk region in slugs and strongly in spores very late in fruiting body formation (Fig. 8e). Of the seven putative stalk genes, three - *DDB_G0280217*, *DDB_G0295797* and *sigK -* were already expressed at low levels in mounds and in the anterior 25–30% of slugs. During culmination these genes became expressed in the stalk and upper- and lower cup, resembling the expression pattern of the prestalk gene *ecmA* [38]. Of the remaining four stalk genes, three - *DDB_G0287091, DDB_G0279361* and *expl7* - are first expressed weakly at the tip or core of the tip and strongly in the stalk, similar to the stalk gene *ecmB* [38]. However, while *ecmB* is also expressed in upper- and lower cup during early culmination, cup expression appears only very late for *DDB_G0287091* and *DDB_G0279361* and not at all for *expl7.* Like *ecmB*, *DDB_G0279361* and *expl7* are also expressed in the basal disc. The remaining gene, *DDB_G0269904*, is only expressed late in the upper stalk.

## Discussion

The major aim of this study was to gain more insight into the nature of cup cells. These cells remain amoeboid during development and show a characteristic localization cradling the spore head. Regarding developmental origins, they are derived from a subpopulation of prestalk cells called prestalk B (pstB) cells and anterior like cells (ALCs), which are prestalk-like cells scattered in the prespore region of slugs, that transdifferentiate from prespore cells. At the onset of fruiting body formation these ALCs and pstB cells sort towards the front and back of the prespore region, while expressing the prestalk genes *ecmA* and *ecmB* from specific regions of their promoters [38, 39] and are supposed to aid in lifting the spore head [10].

A recent RNA-Seq study of a null mutant in diguanylate cyclase, which synthesizes the stalk inducing factor c-di-GMP, identified several cup-specific genes [14], which were first expressed very late in fruiting body formation, when the spore mass has already ascended the stalk. This suggested that the cup cells also have other roles in the fruiting body and that the late cup cells are distinct from the early ALC or pstB derived cup cells, which were found to end up mostly in the stalk [39] and basal disc [40]. We tried to explore this issue using the RNA-Seq data.

### The relationship of cup cells to stalk and spore cells

Both phylogenetic and cluster analysis of RNA-Seq data from purified cell types show that the cup cells are closer to mature stalk cells than to mature spore in terms of their repertoire of expressed genes (Fig. 2). This result is consistent with earlier knowledge about the developmental origin of cup cells from pstB cells and ALCs, which are phenotypically similar to prestalk cells. This conclusion is further substantiated by the observation that the late cup gene *beiB* is expressed in the same cells in the spore head as the prestalk gene *ecmB,* but in a different population than the prespore gene *cotC* (Fig. 7). However, some prespore genes, such as *cotB*, *cotE*, and *pspA*, are expressed at higher levels in cup cells than in spores and stalk cells (Fig. 6b). It is possible that these, but not other spore coat proteins, have a specific role in cup cells. In mature spores, the expression of spore coat genes is actually quite low, likely because they are no longer needed once the spore wall has formed.

### Spore, stalk and cup cell functions as inferred from GO term enrichment and KEGG pathway analysis

GO enrichment analysis of cup specific genes revealed upregulation of genes involved in signal transduction, particularly small GTP-binding proteins, and ubiquitin-mediated proteolysis, but no enrichment of particular metabolic pathways, suggesting that in addition to the proposed supporting role, cup cells also have a signalling role in the spore head. It is worth noting that many of these signalling genes, such as *rapA*, *racE*, and *gpaB* have roles in cell motility and adhesion [41–43]. This may be related to observations that cup cells acquire their upper and lower cup positions by sorting, with adhesion possibly facilitating the formation of the characteristic “cup” shape.

Stalk cells are enriched in autophagy genes and pathways, reflecting that these cells catabolize most of their cell contents. They also contain many extracellular matrix related genes, particularly a large number of uncharacterized proteins with cellulose binding domains, which likely participate in formation of the cellulose stalk wall. Spores express many genes with signalling receptor activity, including many histidine kinase genes. Histidine kinases, such as *dhkA* and *dhkB* are known to play important roles in spore differentiation [44–46]. Our data suggest the involvement of other uncharacterized dhk genes in spore maturation or germination.

### Cell type specific genes show heterogeneity in peak expression

Cluster analysis of the expression profiles of spore, cup and stalk specific genes, retrieved from published data [16] showed that expression of a large proportion of spore, cup and stalk genes increased soon after starvation to reach a peak (cup, spore) or plateau (stalk) at 8 h of development when aggregation is just completed. Other peaks of expression occurred for all three cell types in slugs (16 h), and for stalk genes at 20 h. Peak expression at early stages implies that transcripts decreased before the cells overtly differentiated, which was also evident from the relatively low fraction of prestalk and prespore specific transcripts identified in slugs that were also expressed as stalk and spore transcripts in the mature fruiting body (Fig. 6). One obvious reason is that many prespore and prestalk proteins involved in e.g. spore or stalk wall synthesis are no longer required once the spores and stalk cells have matured. A second reason for poor retention of other prespore and prestalk-enriched proteins could be that they have roles in the motile slug that are essential for slug function rather than spore or stalk cell function.

In addition to genes that are expressed many hours before terminal differentiation, all three terminal cell types also express a significant proportion of genes only at the final stage of maturation. Some of these late expressed genes have been well characterized, although many have unknown functions. In stalks, various cellulose-binding domain-containing proteins, including *staA* and many uncharacterized genes, are expressed late. In spores, the germination factors *celA*, *celB*, *gerA*, *gerB*, and *gerC* are expressed exclusively during terminal differentiation, consistent with a previous study [47]. Another late spore gene, *iptA*, encoding isopentenyl-transferase is required for synthesis of the cytokinin discadenine, a signal molecule that acts both as a germination inhibitor and a sporulation inducer [44, 48].

### Cup cells express extremely high levels of closely related hssA/7E/2C genes

Among the genes that are upregulated very late in cup cells, closely related genes of the hssA/7E/2C gene family are especially abundant. This family consists of nearly 100 genes in *D. discoideum.* Overexpression of both in frame and frame-shifted *hssA* suppresses the culmination defect of a mutant in which statA is partially active, indicating that the *hssA* transcript causes this effect. *HssA* is like *statA* expressed in pstA cells, which take up the front of the prestalk region [24]. 7E and 2C are cAMP-inducible and non-cell-type-specific [49], while *sigN* genes, divided into six group 1 and seven group 2 genes are, like *hssA,* expressed in prestalk cells in an *srfA*-dependent manner. Antibodies raised against group 2 *sigN* genes caused disaggregation of mounds, suggesting that here protein function is relevant [50]. The cup-expressed hssA/7E/2C genes form a separate tight clade that is only expressed in the late cup (Additional file 1: Figure S4). Their expression levels are much higher than those of other members of the family, suggesting a role in cup differentiation that requires further study.

### Novel cell type specific marker genes

Our RNA-Seq dataset of terminally differentiated cells allowed us to establish novel marker genes for terminally differentiated cells (Additional file 1: Table S6). Among the putative cup genes, *DDB_G0276687* (*beiE*) and *DDB_G0271780* (*beiF*) showed, similar to *beiA* and *beiB* [14], only late cup expression, while *tgrR1* was also expressed earlier in slugs and in the stalk. Two spore marker genes, *DDB_G0288489* (*spoA)* and *DDB_G0285289 (spoB)* are similar to another spore marker *spiA* [51] expressed almost exclusively in terminally differentiated spores. Several genes that were deemed stalk-specific from the RNA-seq analysis, such as *DDB_G0287091 (staE)* and *DDB_G0279361 (staF)* also showed cup expression very late in culmination, similar to *staC* [14]. Others such as *sigK*, *DDB_G0280217* and *DDB_G0295797* showed a more *ecmA* like pattern [38] with expression in the slug anterior quarter and early cup. Of the remaining two genes *expl7* was expressed in the stalk and basal disc and and *DDB_G0269904 (staG)* only in the stalk. The expression of stalk genes in the late cup and vice versa is one of the causes underpinning the observed close relationship between the stalk and cup transcriptomes (Fig. 2).

Maeda and coworkers describe considerable heterogeneity in the expression patterns of genes that are enriched in prestalk/ALC cells isolated from slugs, with many genes being expressed in the early upper and/or lower cup, or more exclusively at the tip or the tip inner core [52]. However, their study did not capture any of the late cup and stalk genes reported here. The heterogeneity in (pre) stalk gene expression found in both studies likely reflects that the anterior prestalk cells not only prepare themselves for stalk cell differentiation, but also perform a concerted effort to synthesize the cellulose stalk tube, provide most of the motive force of the slug and are the source of signals that organize morphogenetic movement. The genes involved in these different functions are unlikely to be uniformly regulated by the same signals.

The novel cell-type marker genes will be useful in future studies of the regulation and evolution of terminally differentiated cell types.

## Conclusions

In this study, we collected and analysed the RNA-Seq data of cup cells for the first time, along with that of mature spores, stalks, and vegetative cells. The transcriptome of cup cells was most similar to that of stalks, reflecting the shared cell lineage history between cup and stalk cells. Nevertheless, comparison of the repertoires of cup and stalk specific genes reveals very different roles for these cell types. Cups show overrepresentation of transcripts involved in taxis, motility and signal transduction, especially those encoding small GTPases, implying high signalling and chemotactic activities in cup cells at late development, while autophagy and cell wall synthesis associated transcripts are the hallmark of stalk cells.

Analyses of cell type specific gene expression patterns together with developmental gene expression profiles revealed complex temporal expression dynamics of genes which end up being expressed in mature cell types. Also, it revealed groups of cell type specific genes which are expressed exclusively at late development. Many of these genes have no known functions and the regulation of their expression remains yet to be studied. The data presented in this study, including several newly identified terminal cell type markers, will serve as an important resource for the future study of terminal cell differentiation in *D. discoideum*.

## Methods

### Cell culture and development

*Dictyostelium discoideum* AX2 (gift of G. Gerisch, Max Planck Institut für Biochemie, Martinsried, Germany) was cultured in HL5 medium (Formedium) at 22 °C. For multicellular development, cells were harvested in exponential phase, washed twice with KK2 buffer (16 mM KH_2_PO_4_, 4 mM K_2_HPO_4_, pH 6.5), and plated at 3 × 10^6^ cells/cm^2^ on KK2 agar plates (1.2% Bacto-agar in KK2). Plated cells were incubated at 22 °C for 24 h, at which point the fruiting bodies were collected for further isolation of developmental cell types as described below.

### Cell isolation and RNA-Seq data collection

Vegetative cells grown in shaking HL5 culture were harvested in the exponential phase and RNA was isolated with the RNeasy Mini Kit (Qiagen). Cup cells were isolated using Fluorescent Activated Cell Sorting (FACS). Specifically, a gene fusion of YFP and the promoter of the late cup-specific gene DDB_G0278537 [13] was transformed into *D. discoideum* AX2 strain cells. DDB_G0278537 was initially named *cupB*, but as the CUP acronym was already used for Ca^2+^-upregulated genes, we renamed it *beiB*, after the Chinese word for cup. Cells transformed with [*beiB*]*:YFP* were developed for 24 h into fruiting bodies, which were collected and dissociated in 0.01% TritionX-100 in KK2 and sieved through nylon mesh with 50 μm pore size to remove stalks. Except during enzyme treatment at 22 °C, all cell types were kept at 4 °C up to RNA isolation. The cells in the flow-through were further dissociated by resuspension in pronase/EDTA (1 mg/ml pronase and 50 mM EDTA in KK2) and passing repeatedly through a 23G needle. Cells were then pelleted, re-suspended at 10^7^ cells/ml in 1% BSA in KK2, and sorted with a BD Influx cell sorter (Becton Dickinson). Sorted YFP positive cells were pelleted and RNA was isolated with TRIzol (Thermo Fisher).

To isolate spores, 24 h fruiting bodies were collected, dissociated and sieved through 50 μm nylon mesh as described above. The flow-through containing spores was passed next through 35 μm nylon mesh, and incubated with 0.1% Triton X-100 for 5 min to remove amoeboid cells. Stalk cells were isolated from 24 h fruiting bodies by pipetting vigorously in pronase/EDTA, followed by deposition on 50 μm nylon mesh and extensive washing with KK2. The stalks retained on the mesh were then collected, placed on top of 5 ml 25% Percoll in KK2 and centrifuged at 10,000 g for 5 min at 4 °C. Stalks were collected from the top layer, washed with 5 volumes of KK2 and pelleted at 4000 × g for 3 min. Isolated spore cells and stalks were each placed in RNA lysis buffer with an equal volume of sterile glass beads and vortexed for 15 min at 2500 rpm, followed by RNA extraction using the RNeasy Midi Kit.

The qualities of the RNAs isolated in three independent experiments were assessed with TapeStation (Agilent) to be good (RIN > 7.5) and cDNA libraries were prepared using the Truseq Stranded mRNA Library Prep Kit (Illumina) with Low Sample Protocol. 75-bp paired end reads were sequenced with Illumina NextSeq 500 at the Tayside Centre for Genomic Analysis in two independent runs.

### RNA-seq data analysis

The qualities of RNA-Seq reads were inspected with FastQC [53]. The RNA-Seq reads were then mapped to the *D. discoideum* genome (version 2.7) using Tophat2 [54] with “–b2-very-sensitive” option. A python framework HTSeq [55] was used to count the number of reads mapped to a gene feature. The read counts were then normalized to Transcripts Per Million (TPM) using a previously described formula in order to adjust for the bias due to the difference in the library size and the gene length [56]. Hierarchical clustering was performed and bootstrap values for each cluster were obtained using the R package “pvclust” [57].

Maximum parsimony phylogenetic reconstruction was performed with PAUP after transforming all RNA-Seq data into binary expressions with a threshold of TPM = 3 [58]. A heatmap was drawn with the R package “pheatmap”. The R package “edgeR” was used for differential gene expression analyses [59]. Specifically, genes with the sum of TPM values in all samples less than 3 are filtered out, and the remaining genes were tested for being expressed significantly more than 2-fold in one cell type over the others. Vegetative cells were compared to all the other cell types, while cup, stalk and spore were compared to stalk+spore, cup+spore, and cup+stalk, respectively. The TPM values of all genes and the results of differential gene expression analysis with edgeR are presented in Additional file 2: RNAseqAnalysis.xlsx.

To identify GO terms enriched in each cell type specific gene set, an R package topGO was used with the “classic” algorithm and the Fisher’s exact test [60] against the GO association file, which was downloaded from dictyBase (http://dictybase.org/). Previously published data [16] were used to examine developmental gene expression profiles and prespore/prestalk enrichment of cell type specific genes.

### DNA constructs and cell transformation

Many constructs of cell-type specific promoters fused to either LacZ, YFP or RFP reporter genes were made in the course of this work. The promoter coordinates and oligonucleotide primer sequences of these constructs are listed in Additional file 1: Table S1, together with the restriction sites used in cloning. All sequences were amplified from AX2 genomic DNA using MyTaq (Bioline) or KOD (Merck) DNA polymerase. Plasmid pDdgal-17 [61] was used for all *LacZ* constructs, and pDV-CYFP [62] and mRFPmars [63] for the YFP and RFP constructs, respectively. For *LacZ* constructs, the amplified promoter sequences were cloned into the unique XbaI and BglII sites of the plasmid, fusing the start codon and usually the first few amino-acids of the inserted gene (see Additional file 1: Table S1) in frame with the *LacZ* coding sequence. The primers included XbaI or BglII sites for cloning, or alternatively NheI or BamHI sites, when the promoter sequence contained XbaI or BglII sites, respectively. All constructs were validated by DNA sequencing and transformed into *D.discoideum* AX2 by electroporation. Transformants were selected at 20 μg/ml G418, which was raised to 200 μg/ml G418, when cells expressed the reporter genes only weakly.

### Detection of reporter gene expression

Cells transformed with YFP or RFP constructs were developed on a thin agar, and a small portion of the agar was excised for observation at each developmental stage. Confocal microscopic images were obtained with the Leica TCS SP8 platform (Leica Microsystems), and the images were processed and analysed with Fiji [64].

Cells transformed with *LacZ* constructs were plated at 1–3 × 10^6^ cells/cm^2^ on 2 × 2 cm nitrocellulose filters, supported by non-nutrient agar and developed until the desired developmental stage was reached. Filters were transferred to Whatman 3MM Chromatography paper soaked in 0.5% glutaraldehyde and incubated in a sealed chamber for 3 min. Structures were next fully submersed in 0.5% glutaraldehyde for 3 min. After washing with Z-buffer, structures were stained with X-gal as described previously [65]. The staining periods ranged from 10 min to 24 h, depending on the level of *LacZ* expression, but different developmental stages for the same *LacZ* construct were always stained for the same length of time.

## Additional files

Additional file 1: Supplementary **Figures S1-S4**, Supplementary **Tables S1-S6**. (PDF 1828 kb)

Additional file 2: RNAseq analysis. (XLSX 10006 kb)

Additional file 3: GO analysis. (XLSX 924 kb)

Additional file 4: KEGG analysis. (XLSX 68 kb)

Additional file 5: k means cluster analysis. (XLSX 753 kb)
