# Supplementary material for: Cell-type specific RNA-Seq reveals novel roles and regulatory programs for terminally differentiated Dictyostelium cells
Source: BMC Genomics. 2018 Oct 22;19:764. doi: 10.1186/s12864-018-5146-3 (PMC6198379; doi:10.1186/s12864-018-5146-3)
Supplement: Supplementary file 1 — Supplementary Figures S1-S4, Supplementary Tables S1-S6. (PDF 1828 kb) [file 12864_2018_5146_MOESM1_ESM.pdf]

Supplementary figures S1-S4, Supplementary tables S1-S6

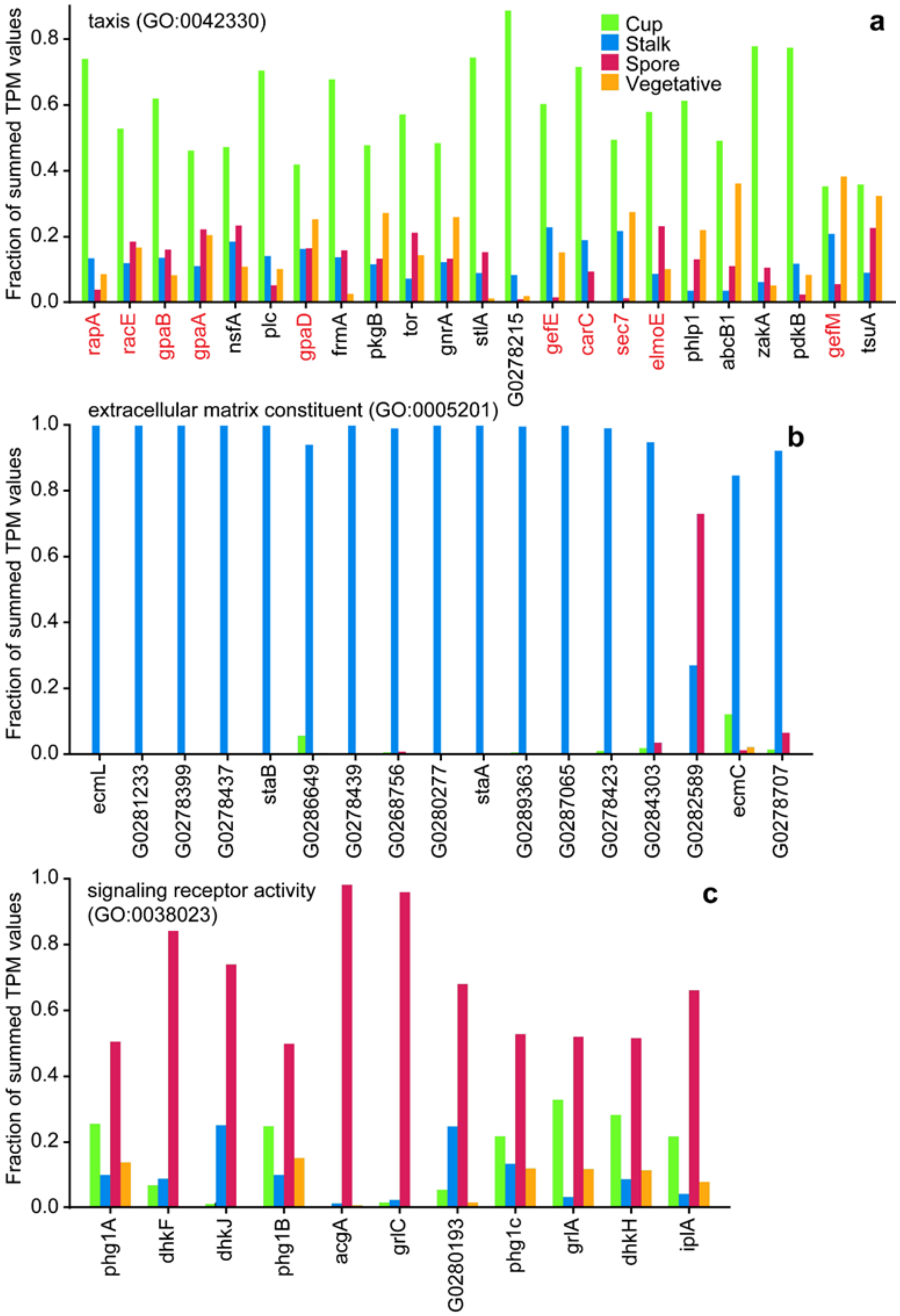

Figure S1: Cell type specific expression of genes associated with representative GO terms. TPM values in cup, stalk, spore, and vegetative cells are presented as fraction of the summed values for the four cell types. **a** Cup specific genes are overrepresented with genes associated with “taxis”. Many of these (in red) are also associated with “small GTPase mediated signalling”. **b** Stalk specific genes are overrepresented with genes with cellulose binding domains annotated as “extracellular matrix structural constituent. **c** Spore specific genes include many genes with “signalling receptor activity”. DDB_ prefixes were removed from gene identifiers.

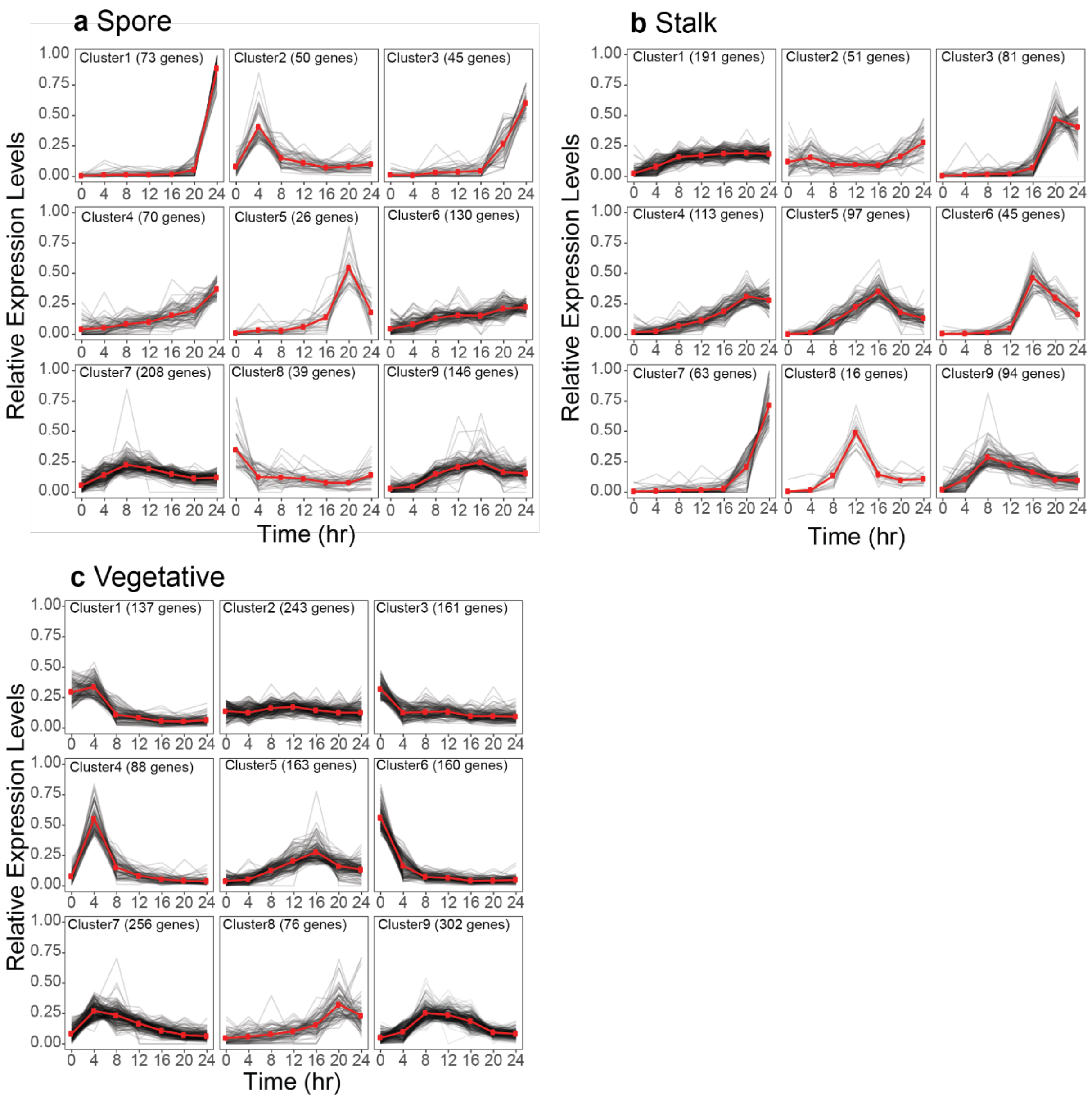

Figure S2: k-means cluster analysis of **a** spore, **b** stalk and **c** vegetative cell specific genes. The developmental expression profiles of spore, stalk, and vegetative cells were subdivided into 9 clusters by k-means clustering. The number of genes which belong to each cluster is shown in parentheses. The average trend is shown in red points and lines.

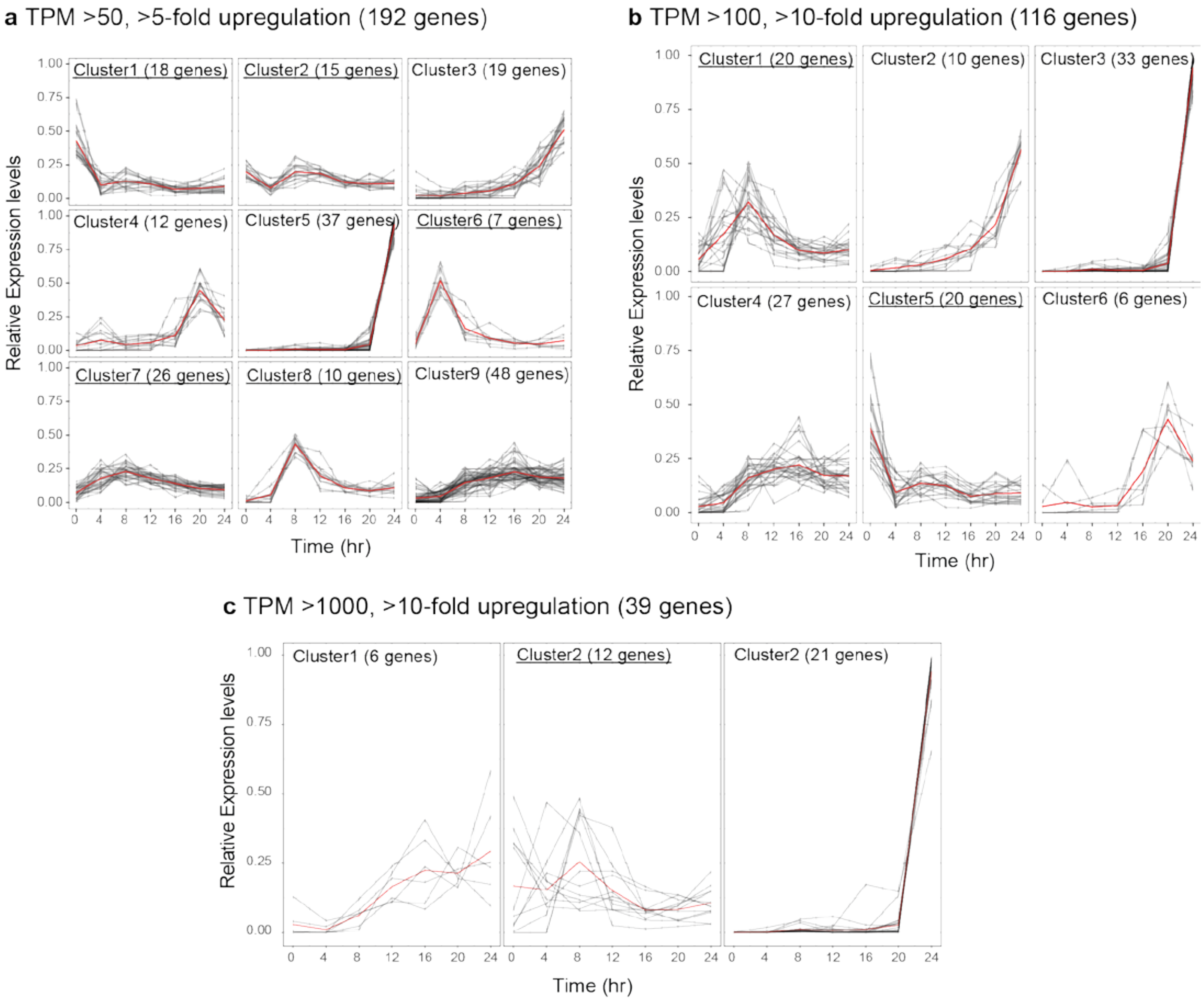

Figure S3. k-means cluster analysis of cup cell specific genes with varying thresholds. The robustness of the overall patterns of gene expression, especially the presence of genes with early peaks, was tested by making a more stringent set of cup cell specific genes. We set the following thresholds: **a** expression levels with more than 50 TPM and more than 5-fold upregulation relative to the average of the expression levels of spore and stalk (192 genes met this criterion); **b** expression levels above 100 TPM and more than 10-fold upregulation (116 genes); **c** expression levels above 1000 TPM and more than 10-fold upregulation (39 genes). We set smaller number of k-means clusters for b and c to avoid unnecessary fine subdivisions of clusters. Note that even in more stringent sets of cup cell specific genes, there exist 30-40% of genes with early expression peaks. Clusters which we regard as containing “early peak” genes are underlined. The number of genes which belong to each cluster is shown in parentheses. The average trend is shown in red points and lines.

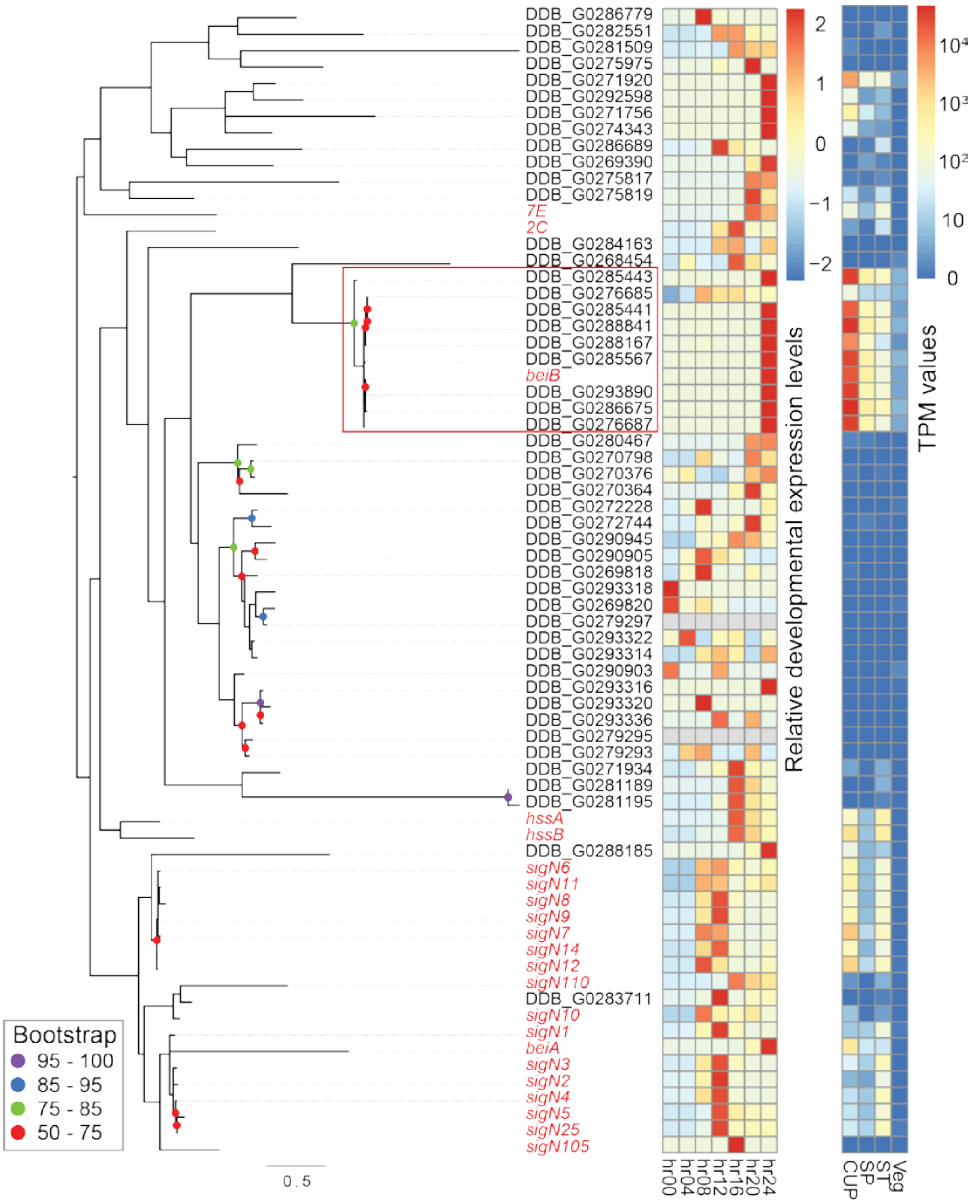

Figure S4: Phylogenetic tree of hssA/2C/7E genes presented with the heat maps of their developmental profiles as reported by Parikh et al. [16], and cell type expression profiles as determined in this study. Amino acid sequences of genes annotated as “hssA/2C/7E related genes” were collected from dictyBase (http://dictybase.org/), aligned with muscle (https://www.drive5.com/muscle/) and reconstructed with RAxML under the WAG+Γ model. Bootstrap values are presented by the colour of circle on each internal node as shown in the legend at the bottom left. Genes reported previously are shown in red italics, and a closely related group of genes with very high expression levels in cup cells is emphasized by a red rectangle. The heat map of developmental expression profiles was scaled by row and the relative expression levels are shown. The heat map of gene expression levels in each cell type is not scaled by row and TPM values on log10-scale are presented.

Table S1: Promoter coordinates and oligonucleotide primer sequences of constructs used in the present study.

Gene ID	coordinates from start	5' gene	R.sites	primer	DNA sequence
G0280217	-1271_+3	-1472	XbaI	280217NF	CCC**TCTAGA**GATTTGTATTTTTCATTTTTCATTTTATGG
			BglII	280217NR	CCC**AGATCT**CATTCTTCCTTTAATTAATTATTGTAATGG
G0269904	-1555_+160 intron +13_+104	-1519	XbaI	269904NF	CCC**TCTAGA**ATTGGCTTCATTAAAAATATAAATTCTTC
			BamHI	269904NF	CCC**GGATCC**TGACTTATTTGTTTGGGTTGATTTAAC
G0288489	-743_+27	-709	XbaI	288489F	CCC**TCTAGA**TTGCTATAAACATAAAATTAATCCGG
			BglII	288489R	CCC**AGATCT**ATATTCTCGTGCATACATTGTTGAC
G0285289	-851_+12	-710	XbaI	285289F	CCC**TCTAGA**GCTTCGTCTGTTGTACCACCAG
			BamHI	285289R	CCC**GGATCC**TTGTGATGTCATTTTTAAAAAATATTTATTTA C
G0287091	-718_+6	-649	XbaI	287091F	CCC**TCTAGA**GATCCAAGTGAAATTTTAAAGTATTCTTTAA G
			BamHI	287091R	CCC**GGATCC**CTCCATTTTTTTTTATTTTAATTATTTCAATT TAG
G0295797	-808_+3	-807	XbaI	295797F	CCC**TCTAGA**GTCTTTGATGTTTTCTAATGAACAATAGTG
			BglII	295797R	CCC**AGATCT**CATTTTTTTTTTTTTTTTTTTTTAATTTATTTAT ATTTATATTTG
G0276687	-892_+9	-830	XbaI	276687F	CCC**TCTAGA**AGCTCAAAAAACAAATCAACCTG
			BglII	276687R	CCC**AGATCT**TAGGGTCATTTTAAAATTTTAATAGTTTCTT G
G0271780	-1072_+9	-1034	NheI	271780F	CCC**GCTAGC**CGATGCTAGATTTAGACATGTAG
			BglII	271780R	CCC**AGATCT**ATCTTCCATTTTTTAATATCAATTG
SigK	-632_+24	-597	XbaI	SigKF	G**TCTAGA**AATTAAATTAATGAAACCAATAG
			BglII	SigKR	**AGATCT**TGATAAAATTTTCATTTTTTTCATTTC
Expl7	-1225_+39	-1177	XbaI	Expl7F	**TCTAGA**GAAGATGAAGGTTTTGAAATGG
			BglII	Expl7R	**AGATCT**TAAAGAAAGTGTTAAAATTAAACTACC
G0279361	-980_+96	-931	XbaI	361F	**TCTAGA**GGAGAAATCACTATTAGTTGCTG
			BglII	361R	**AGATCT**TGAATCAACAACTGTATCAATACCTTC
tgrR1	-925_+56	-857	BamHI	tgrR1F	**GGATCC**CAAAATATTGGCACAATAGTAAAAAC
			BglII	tgrR1R	**AGATCT**CTTCAAGACAACCATGTTC
cotC	-743 to stop	-1492	XbaI	cotC-f	**TCTAGA**CCCATACTACATTAAAATATTTGTATATC
			BglII	cotC-r	**GGATCC**ATAGTCCCATTCATCATTTGC
G0278537	-930_+77	-931	SalI	278537prF	AAA**GTCGAC**ACATCTTGTTCTTTTTCAAGCT
			BglII	278537prR	ATT**AGATCT**TCTTGAATATTGGCTGATGTCAATG

Primers used to amplify promoter sequences for fusion to LacZ, YFP and RFP reporter genes. The coordinates of the amplified promoter relative to the start codon, the position of the gene upstream from the target gene start codon and the restriction (R) sites used for cloning are shown in columns 2-4.

Table S2: RNA-Seq reads alignment statistics.

Sample	Total input reads	Total mapped reads	Overall mapping rate (%)	Concordant pair alignment rate (%)
Cup1	28963740 (24379521)	21221347	73.3 (87.0)	69.8 (83.0)
Cup2	28240290 (24777269)	21749307	77 (87.8)	72.1 (82.2)
Cup3	25184474 (21977922)	19276490	76.5 (87.7)	71.8 (82.3)
Stalk 1	42229572	39346131	93.2	87.8
Stalk 2	28434400	26739558	94	89.3
Stalk 3	29557000	27773670	94	89.7
Spore 1	23808942	22455428	94.3	91
Spore 2	26669622	25214792	94.5	91.4
Spore 2	29245912	27445429	93.8	90.1
Vegetative 1	26161188	24486859	93.6	88.1
Vegetative 2	35515914	33461322	94.2	89.9

The paired-end RNA-Seq reads obtained in this study were all mapped with Tophat2 to the genome of *D. discoideum*. Total number of input reads, the number of reads successfully mapped to the genome, the overall mapping rate, and the concordant pair alignment rate are shown for each sample. The numbers in parentheses in cup cell samples represent the statistics when reads mapping to YFP are removed.

Table S3: Transcription factors upregulated in cup cells

Gene Name	Gene products	Cup	Stalk	Spore	Fold-change
srfA	MADS-box transcription factor, SRF-related protein	2464	1532	134	3.0
mybE	myb domain-containing protein	903	52	164	8.4
stkA	GATA-binding transcription factor	635	387	79	2.7
bzpH	putative basic-leucine zipper (bZIP) transcription factor	146	27	20	6.2
G0280723_ps	pseudogene	104	39	9	4.4
G0286351	putative transcriptional regulator	101	40	0	5.0
G0278761	NF-X1-type zinc finger-containing protein	94	4	14	10.6
G0284255	C2H2-type zinc finger-containing protein	85	33	27	2.9
bzpD	putative basic-leucine zipper (bZIP) transcription factor	82	32	16	3.4
mybG	myb domain-containing protein	77	10	54	2.4
dstB	signal transducer and activator of transcription (STAT)	60	29	15	2.7
G0272048	C2H2-type zinc finger-containing protein	45	6	2	10.5
hbx13	homeobox transcription factor Hbx13	36	10	1	6.7
G0287317	putative transcriptional regulator	34	20	4	2.8
hbx9	homeobox transcription factor Hbx9	32	17	3	3.3
mybL	putative myb transcription factor	29	25	6	1.9
taf11	transcription initiation factor TFIID subunit	24	3	2	8.9
bzpE	putative basic-leucine zipper (bZIP) transcription factor	24	5	17	2.2
gtf2h2	TFIIH subunit, general transcription factor IIH component	22	8	3	3.9
lsrA	Nulp1-type basic helix-loop-helix domain-containing protein	17	1	3	7.6
G0268502	C2H2-type zinc finger-containing protein	17	1	0	43.9
rpa12	RNA polymerase I subunit	15	0	1	21.9
mybN	putative myb transcription factor	11	5	1	3.4
gtf3C5	transcription factor IIIC-epsilon subunit	8	4	2	2.7
gtaX	putative GATA-binding transcription factor	7	1	1	12.0
G0278995	C2H2-type zinc finger-containing protein	5	0	1	7.8
mybU	myb domain-containing protein	4	1	0	8.3
G0269884	zf-C2HC5 zinc-finger	1	0	0	6.6
tfiiiA	C2H2-type zinc finger-containing protein, transcription initiation factor IIIA	1	0	0	4.6

For all tables S3-S5 DDB_ prefixes were removed from gene names

Table S4: Transcription factors upregulated in stalks

Gene Name	Gene products	Cup	stalk	spore	Fold-change
G0291348	fungal transcriptional regulatory protein, putative zinc cluster transcription factor	325	1229	4	7.5
bzpF	putative basic-leucine zipper (bZIP) transcription factor	402	902	9	4.4
gtaI	putative GATA-binding transcription factor	448	664	49	2.7
gtaG	putative GATA-binding transcription factor	250	472	3	3.7
G0275571	Arf GTPase activating protein	35	248	189	2.2
gtaC	GATA-binding transcription factor	59	243	6	7.5
cudA	transcriptional regulator CudA	129	191	5	2.8
G0284591	C2H2-type zinc finger-containing protein	95	164	16	3.0
mybK	myb domain-containing protein, SSXT family protein	11	90	55	2.7
cbfB	putative C-module-binding factor, transcription factor jumonji	1	86	1	99.9
hbx14	homeobox transcription factor Hbx14	5	66	0	26.0
bzpL	putative basic-leucine zipper (bZIP) transcription factor	10	33	6	4.1
jcdJ	C2H2-type zinc finger-containing protein, transcription factor jumonji,	6	31	17	2.6
bzpO	putative basic-leucine zipper (bZIP) transcription factor	7	28	11	3.1
tacA	transcription factor TacA	2	20	8	3.7
G0272672	C2H2-type zinc finger-containing protein	2	19	5	5.2
mybB	putative myb transcription factor,	5	18	9	2.7
G0270306	putative transcriptional regulator	1	15	1	15.1
mybD	myb domain-containing protein	1	8	0	10.9
G0270590	fungal transcriptional regulatory protein, putative zinc cluster transcription factor	1	5	2	3.5
G0278963	Transcription factor S-II	1	5	4	2.4
G0272264	coiled-coil domain-containing protein	0	1	0	5.0
G0282499	transcription factor E2F/dimerisation partner (TDP) family protein	0	1	0	23.3

Table S5: Transcription factors upregulated in spores

Gene Name	Gene products	Cup	stalk	spore	Fold-change
dr1	putative histone-like transcription factor	15	37	402	15.6
G0278179	myb domain and DNAJ heat shock N-terminal domain-containing protein	128	48	354	4.0
mrfA	transcription factor MrfA	211	32	297	2.4
G0274691	C2H2-type zinc finger-containing protein	57	12	220	6.4
G0271886	PHD zinc finger-containing protein, bromodomain-containing protein	16	9	165	13.0
gtaH	putative GATA-binding transcription factor	31	38	148	4.3
hbx10	homeobox transcription factor Hbx10	6	1	91	25.1
gtaN	putative GATA-binding transcription factor	1	2	79	45.0
gtaE	putative GATA-binding transcription factor	2	60	73	2.4
cdc5l	myb domain-containing protein	9	8	61	7.0
G0267638	Myb, SNF2-related, CHR group and helicase, C-terminal domain-containing protein	7	10	59	6.8
G0285493	Bromo domain and TAF8-C domain containing protein	0	2	49	48.7
drap1	putative histone-like transcription factor	17	6	46	4.1
mybX	myb domain-containing protein	14	4	46	5.2
G0292186	NDT80/PhoG-like protein	11	3	41	5.9
swi3	SWIRM domain-containing protein Swi3	15	4	40	4.2
G0271948	YEATS family protein	6	2	28	7.4
G0269466	BSD domain-containing protein	9	2	24	4.2
G0269842	C2H2-type zinc finger-containing protein	5	7	24	4.0
G0286135	myb domain and ZZ-type zinc finger-containing protein	1	1	24	37.1
isw	Myb, SNF2-related, ATP-dependent chromatin-remodelling factor	3	4	24	6.2
mybS	myb domain-containing protein	2	5	21	6.1
bzpS	putative basic-leucine zipper (bZIP) transcription factor	1	1	18	18.2
G0272740	putative histone-like transcription factor	4	2	17	5.6
mybW	myb domain-containing protein	3	1	13	7.9
wrky1	putative WRKY transcription factor	0	2	10	7.5
taf6	TATA-binding protein-associated-factor	1	1	10	9.0
mf12	C2H2-type zinc finger-containing protein	1	1	9	10.7
gtaU	putative GATA-binding transcription factor,	1	2	9	6.5
rbbB	putative Retinoblastoma (Rb) binding protein	1	2	7	5.0
gtf2h4	general transcription factor IIH, polypeptide 4, TFIIH subunit	2	1	7	5.0
jcdG	jmjC domain-containing protein, cupin region-containing protein	0	1	5	15.0
mybY	myb domain-containing protein	1	1	5	4.5
ybl1	YB-like 1, DNA polymerase epsilon p17 subunit 3	0	1	4	13.8
taf9	transcription initiation factor TFIID subunit,	0	1	4	5.8
G0280121	C2H2-type zinc finger-containing protein	0	0	2	7.3
G0268506	putative histone-like transcription factor	0	0	2	9.0

Table S6. Novel marker genes for terminally differentiated cells

**Cup specific**, Chen et al., 2017 [14]	duplicate name	new name
DDB_G0276063	*cupA*	*beiA*
DDB_G0278537	*cupB*	*beiB*
DDB_G0282455	*cupC*	*beiC*
DDB_G0293854	*cupD*	*beiD*
**Cup specific**, this study		
DDB_G0276687		*beiE*
DDB_G0271780		*beiF*
**Spore specific**		
DDB_G0288489		*spoA*
DDB_G0285289		*spoB*
**Stalk specific**		
DDB_G0287091		*staE*
DDB_G0279361		*staF*
DDB_G0269904		*staG*
